# Left atrial appendage morphofunctional indices could be predictive of arrhythmia recurrence post-atrial fibrillation ablation: a meta-analysis

**DOI:** 10.1186/s43044-023-00356-3

**Published:** 2023-04-20

**Authors:** Konstantinos A. Papathanasiou, Dimitrios A. Vrachatis, Dimitrios Kazantzis, Charalampos Kossyvakis, Sotiria G. Giotaki, Gerasimos Deftereos, Konstantinos Raisakis, Andreas Kaoukis, Dimitrios Avramides, Vaia Lambadiari, Gerasimos Siasos, Spyridon Deftereos

**Affiliations:** 1 Second Department of Cardiology, National and Kapodistrian University of Athens, Medical School, Attikon University Hospital, 1 Rimini Str., Chaidari, Attiki, 12462 Athens, Greece; 2 Bristol Eye Hospital, University Hospitals Bristol and Weston NHS Foundation Trust, Bristol, UK; 3 Department of Cardiology, “G. Gennimatas” General Hospital of Athens, Athens, Greece; 4 Second Department of Internal Medicine, National and Kapodistrian University of Athens, Medical School, Attikon University Hospital, 12462 Athens, Greece; 5 3rd Department of Cardiology, National and Kapodistrian University of Athens, Medical School, Sotiria Chest Disease Hospital, Athens, Greece

**Keywords:** Atrial fibrillation, Catheter ablation, Arrhythmia recurrence, Left atrial appendage

## Abstract

**Background:**

Left atrium changes are implicated in atrial fibrillation (AF) substrate and are predictive of AF outcomes. Left atrial appendage (LAA) is an integral component of left atrial structure and could be affected by atrial cardiomyopathy. We aimed to elucidate the association between LAA indices and late arrhythmia recurrence after atrial fibrillation catheter ablation (AFCA).

**Methods:**

The MEDLINE database, ClinicalTrials.gov, medRxiv and Cochrane Library were searched for studies evaluating LAA and late arrhythmia recurrence in patients undergoing AFCA. Data were pooled by meta-analysis using a random-effects model. The primary endpoint was pre-ablation difference in LAA anatomic or functional indices.

**Results:**

A total of 34 studies were found eligible and five LAA indices were analyzed. LAA ejection fraction and LAA emptying velocity were significantly lower in patients with AF recurrence post-ablation [SMD = − 0.66; 95% CI (− 1.01, − 0.32) and SMD = − 0.56; 95% CI (− 0.73, − 0.40) respectively] as compared to arrhythmia free controls. LAA volume and LAA orifice area were significantly higher in patients with AF recurrence post-ablation (SMD = 0.51; 95% CI 0.35–0.67, and SMD = 0.35; 95% CI 0.20–0.49, respectively) as compared to arrhythmia free controls. LAA morphology was not predictive of AF recurrence post-ablation (chicken wing morphology; OR 1.27; 95% CI 0.79–2.02). Moderate statistical heterogeneity and small case–control studies are the main limitations of our meta-analysis.

**Conclusions:**

Our findings suggest that LAA ejection fraction, LAA emptying velocity, LAA orifice area and LAA volume differ between patients suffering from arrhythmia recurrence post-ablation and arrhythmia free counterparts, while LAA morphology is not predictive of AF recurrence.

**Supplementary Information:**

The online version contains supplementary material available at 10.1186/s43044-023-00356-3.

## Background

Atrial fibrillation (AF) is the most common arrhythmia in clinical practice affecting 44 million people worldwide, and its incidence is expected to increase further in the following years [1].

AF presence has been associated with adverse long-term outcomes, namely twofold increase in total mortality, heart failure prevalence and hospitalizations, as compared to non-AF patients. What is more, AF course is frequently complicated by systemic embolic events, vascular dementia and impaired quality of life [2].

Anticoagulation, heart rate control and management of comorbidities are the three major therapeutic pillars in the treatment of patients with AF and have been shown to reduce overall mortality, hospital admissions and thromboembolic events [3]. Rhythm control is currently recommended for symptom control and quality of life improvement in symptomatic AF patients, despite optimally tolerated heart rate and comorbidities control. In particular, AF catheter ablation (AFCA) is indicated for rhythm control after one failed or intolerant antiarrhythmic drug has been tested [2].

AF catheter ablation (AFCA) has revolutionized AF management and two recently published trials suggested that cryoablation is more effective first line approach than antiarrhythmic drugs [4, 5]. On the contrary, AFCA has been hindered by a ceiling of long term success rate ranging between 65 and 78% [6]. Importantly, current arrhythmia recurrence prediction scores post-AFCA feature moderate discriminatory ability [7].

2020 European Society of Cardiology guidelines for the diagnosis and management of atrial fibrillation endorsed (class IIa recommendation) a structured characterization of AF (the so-called 4S-AF scheme). Stroke risk, severity of AF burden, symptom status and AF substrate constitute the 4S-AF scheme. Hence, the role of AF substrate is increasingly recognized and non-invasive multimodality imaging is capable of characterizing left atrial (LA) morphology and function, which have been proven so far predictive of stoke development [8] as well as AF recurrence post-AFCA [9].

Since left atrial appendage (LAA) is a critical morphofunctional component of LA, we hypothesized that AF related atrial cardiomyopathy also affects LAA anatomic and functional indices. Previous studies have shown that LAA emptying velocity and ejection fraction are improved three months after catheter ablation in patients with paroxysmal AF [10]. In this systematic review we attempted to answer the question of whether pre-ablation LAA indices could be predictive of AFCA long term success.

## Methods

### Data sources and search strategy

Studies including patients undergoing first catheter ablation for AF in whom late arrhythmia recurrence was assessed were evaluated for inclusion in this meta-analysis. Search strategy, study selection, data extraction, and data analysis were performed according to the Preferred Reporting Items for Systematic Reviews and Meta-Analyses 2009 guidelines [11]. Two reviewers (KP and DV) independently identified the relevant studies by an electronic search of the MEDLINE database, ClinicalTrials.gov, medRxiv and Cochrane Library from inception to 15^th^ of August 2022. The following search query was used: ‘’atrial appendage’’ and ‘’atrial fibrillation ablation recurrence” (see Additional file 1: Table S1). Articles and book chapters cited in the reference lists of initially identified articles by this query were reviewed in order to identify any supplemental studies (“snowball procedure”). The final list of eligible articles was filtered manually to exclude duplicates. No language restriction was utilized, and all relevant studies were screened irrespective of study design (randomized and non-randomized studies of retrospective or prospective design).

### Inclusion and exclusion criteria

In order for a study to be eligible, it had to fulfill the following inclusion criteria: (1) evaluated late arrhythmia recurrence rates in patients undergoing AFCA (2) employed a clearly stated definition of arrhythmia recurrence (3) reported data on pre-ablation LAA indices and described the employed imaging modality and technique. Studies were excluded if they were: (1) not reporting data on arrhythmia recurrence and/or LAA indices (2) case reports (3) evaluating arrhythmia recurrence and/or LAA indices in other ablation modalities for AF (surgical, epicardial or hybrid ablation).

### Data extraction

Data were independently extracted and reviewed from each study by two reviewers (KP, DV). Any discrepancy between data extractions was resolved by discussion or a third reviewer (SG). The following data were extracted: first author, year of publication, country, study design (prospective/retrospective), number of patients, patient demographics, matching criteria and descriptive statistics of recurrence and no recurrence groups, LAA indices and imaging technique, and catheter ablation modality and technique.

### Quality assessment

Quality of the included studies was conducted via the Newcastle–Ottawa Scale (NOS) [12], in which a study was judged on three categories: selection, comparability and exposure/outcome. A nine-point scale of the NOS (range, 0–9 points) was eventually used for the evaluation. Two authors (KP, SG) discussed the implementation of this quality assessment tool and independently assessed the studies. Studies were defined as high quality if they had more than seven points, as medium quality if they had between four and six points, and as poor quality if they had fewer than four points.

### Outcomes of interest

The pre-specified primary endpoint was pre-ablation difference in LAA anatomic or functional indices between patients suffering arrhythmia recurrence and arrhythmia free counterparts post-AFCA. LAA indices were assessed and measured according to the definitions reported in the original study protocols (see Additional file 1: Tables S2 and S3).

### Statistical analysis

The descriptive statistics were described as mean ± standard deviation (SD). For continuous variables the standardized mean difference (SMD) with 95% confidence intervals (CI) was used as the summary statistic and trial-specific data were pooled with the inverse-variance random-effects method. When mean and standard deviation were not available, they were derived from sample size, median, and range based on a method previously described by Wan et al. [13]. For categorical variables statistical pooling was performed according to a random-effect model with generic inverse-variance weighting of odds ratio, computing risk estimates with 95% CI. The presence of heterogeneity among studies was evaluated with the Cochran Q chi-square test with *p* ≤ 0.1 considered to be of statistical significance, estimating the between-studies variance tau-square, and using the *I*^2^ test to evaluate inconsistency. *I*^2^ values of 25%, 50% and 75% were assigned adjectives of low, moderate and high heterogeneity. A leave-one-out sensitivity analysis was performed by iteratively removing one study at a time to confirm that our results were not driven by any single. In addition, a sensitivity analysis by calculating SMD using the inverse-variance fixed-effects method was performed for all outcomes of interest. Publication biases were assessed by visual inspection with funnel plots. All analyses were performed with Review Manager, version 5.3 (Copenhagen: The Nordic Cochrane Centre, The Cochrane Collaboration, 2014). The guidelines summarized in the MOOSE statements were followed [14].

## Results

### Search results

**Fig. 1:**
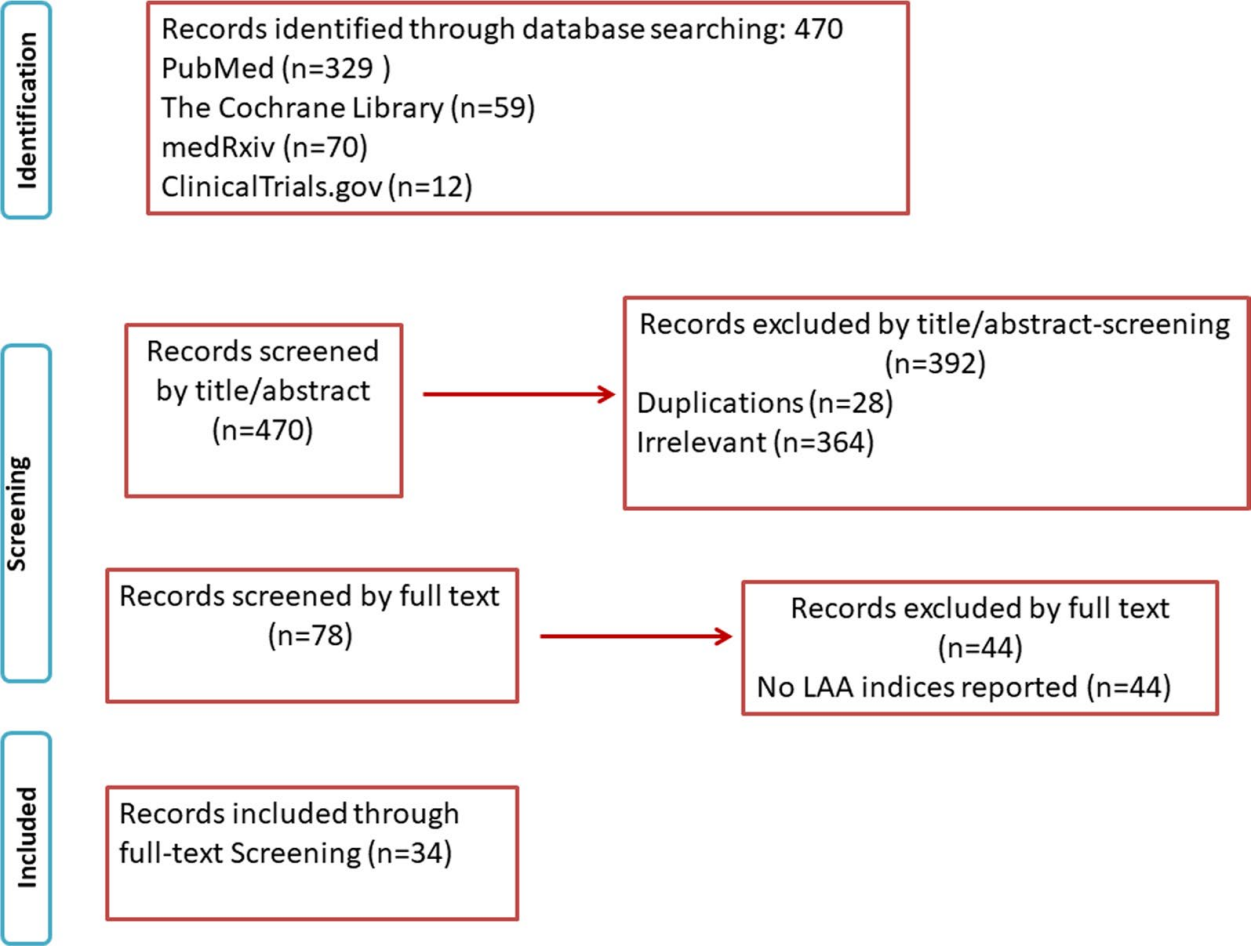
PRISMA flowchart

Figure 1 displays the PRISMA study search and selection process. The electronic database search identified 470 studies. After screening, a total of 34 studies [15–48] met the inclusion criteria. In particular, 5, 11, 6, 5, and 20 studies were included in the LAA ejection fraction (LAAEF), LAA volume (LAAV), LAA morphology, LAA orifice area (LAAOA) and LAA emptying velocity (LAAeV) analyses, respectively. Individual study characteristics are presented in Table1.

**Table 1:** Studies main characteristics

Author/Year	Country	Study type	N	AFCA type	AF type	FU	Age (years)	Female (%)	HF (%)	LAA indices
Tsao [16]	Taiwan	R	68	RF	PAF, Per-AF	228 ± 79 d	53.3 ± 9.0	23.5	N/R	LAAEF
Park [27]	Korea	R	264	RF	PAF, Per-AF	33.2 ± 12.4 mo	54.8 ± 10.8	19.7	16.28	LAAV LAAEF
Machino-Ohtsuka [38]	Japan	R	123	RF	Per-AF	18 ± 2.3 mo	60 ± 9	15	N/R	LAAeV
Yoshida [43]	Japan	R	67	RF	PAF, Per-AF	6 mo	64 ± 8	13.43	N/R	LAAeV
Combes [44]	France	P	40	RF	Per-AF	12 mo	60 ± 11	15	N/R	LAAeV
Kim 2014	Korea	R	130	RF	Per-AF	33 mo (24–52)^+^	54.6 ± 9.3	13.84	30	LAAEF
Gerede [45]	Turkey	P	51	CB	PAF	12mo	54.6 ± 10.4	50.9	23.5	LAAeV
Fukushima [46]	Japan	P	105	RF	PAF	19 mo( 6–38)^+^	57 ± 12	23.5	N/R	LAAeV
Ariyama [47]	Japan	R	41	RF	NPAF	12 mo	58 ± 10	7	9.75	LAAeV
Ma [48]	China	P	120	RF	PAF, Per-AF	12 mo	64 ± 7	40	8.3	LAAeV
Nakatani [20]	Japan	R	126	RF	PAF, Per-AF	12 mo	62 ± 11	22	15	LAAeV
E Gul [18]	Canada	R	59	RF	PeAF	13 mo (4–67)^+^	64.6 ± 9.8	56	N/R	LAAV LAA_m
Zheng [19]	China	P	62	RF	PAF, Per-AF	N/R	N/R	N/R	N/R	LAAV
Shiozawa [17]	Japan	P	77	RF	PAF, Per-AF	580 ± 255 d	59 ± 8	19.48	N/R	LAAV LAAeV LAAOA LAA_m
Pinto Teixeira [21]	Portugal	P	52	RF	PAF, Per-AF	305.9 ± 144.6 d	54 ± 10	42	N/R	LAAV
Nedios [22]	Germany	R	104	RF	PAF, Per-AF	26 ± 14 mo	58 ± 10	31	5.76	LAAV
He [23]	China	P	80	RF	PAF	12 mo	57.31 ± 10.42	40	20	LAAeV LAAEF
Kocyigit [24]	Turkey	P	359	CB	PAF, Per-AF	37 mo (17–60)^+^	56.41 ± 7.88	49.42	N/R	LAAOA LAA_m
Du [25]	China	R	108	RF	PAF, Per-AF	12 mo	63.1 ± 8.1	46.3	N/R	LAAV LAAOA
Tian [26]	China	R	83	RF	PAF, Per-AF	19 mo (4–24)^+^	60.36 ± 10.11	40.9	21.6	LAAV LAAEF
Yang 2020	China	P	215	RF	Per-AF	R( +): 4 mo (3–5)^+^ R(-): 6 mo (6–6)^+^	R( +) 62.9 ± 9.4 R(-) 62.8 ± 9.6	R( +)16.4 R(-) 26.3	N/R	LAAeV
Wei [29]	China	P	150	RF	PAF, Per-AF	14 mo^+^	64 ± 11	45.3	12.6	LAAeV
Straube [30]	Germany	R	473	CB	PAF, Per-AF	19 mo^+^	66.2 ± 9.5	40	N/R	LAAV LAAOA LAA_m
Gong [31]	China	R	84	RF	PAF, Per-AF	618.6 d	R( +) 67.5 ± 7.5 R(-) 66.1 ± 9.0	30.95	N/R	LAAeV LAA_m
Yang [28, 32]	China	R	164	RF	Per-AF	15 mo (12–18)^+^	58.2 ± 9.7	23.17	9.8	LAAeV
You [33]	China	R	238	RF, CB, RF + CB	PAF	48 mo^+^	59.37 ± 11.0	45.4	5.9	LAAeV
Istratoaie [34]	Romania	P	81	RF	PAF	12 mo (11–14)^+^	55.3 ± 9	40.7	N/R	LAAeV
Ma [35]	China	R	124	RF	PAF, Per-AF	12 ± 3 mo	R( +) 65.5 ± 6 R(-) 62.6 ± 7.3	R( +)31.7 R(-) 41.4	N/R	LAAeV
Kielbasa 2021	Poland	R	417	CB	PAF	24 mo (15.5–45.6)^+^	59 (mean)	39.3	3.1	LAAeV
Kim [37]	Korea	R	992	RF	PAF, Per-AF	36 mo	55.8 ± 10.5	20.4	4	LAAV
Kim [39]	Korea	R	3.120	RF	PAF, Per-AF	36 mo	55.74 ± 10.96	21.1	9.4	LAAeV
Spittler [40]	Germany	R	134	RF	Per-AF	19.6 ± 14.6 mo	63.8 ± 10.2	36.6	N/R	LAAeV
Simon [41]	Hungary	R	561	RF	PAF, Per-AF	recurrence-free time 22.7 mo^+^	61.9 ± 10.2	34.9	12.7	LAAV LAAeV LAAOA
Szegedi [42]	Hungary	R	428	RF	PAF	8.8–43 mo	60.7 ± 10.8	35.5	6.1	LAA_m

^+^ = median, R( +) = recurrence, R(-) = no recurrence, *N/R* not reported, *PAF* paroxysmal atrial fibrillation, *Per-AF* persistent atrial fibrillation, *NPAF* non-paroxysmal atrial fibrillation, *AFCA* atrial fibrillation catheter ablation, *RF* radiofrequency ablation, *CB* cryoablation, *FU* follow-up, *N* study population, *R* retrospective, *P* prospective, *mo* months, *d* days, *HF* heart failure, *LAA* left atrial appendage, *LAAV* LAA volume, *LAAeV* LAA emptying velocity, *LAAOA* LAA orifice area, *LAAEF* LAA ejection fraction, *LAA_m* LAA morphology, chicken wing versus non-chicken wing

Nineteen studies included a mixed population of paroxysmal and persistent AF patients, seven studies included only paroxysmal AF patients, and the remaining eight studies included patients suffering from persistent AF. Regarding AF ablation modality, four studies reported cryoablation [24, 30, 36, 45], one study reported a mixed population of cryoablation and radiofrequency ablation [33], and the remaining studies reported only radiofrequency ablation. Of note all studies defined early recurrence as a recurrence of AF within three months post-ablation and late recurrence as the recurrence of AF episodes or other atrial tachyarrhythmias lasting more than 30 s after the three months blanking period. Furthermore, all studies report a follow-up period of at least 6 months post-AFCA and 24-h Holter monitoring at least at 3, 6 and 12 months post-AFCA. As far as antiarrhythmic drug (AAD) post-AFCA is concerned, twelve studies do not report any standard protocol, while most of the remaining studies permitted AAD administration post-AFCA, if patients were already taking them or if they presented with persistent AF subtype. All LAA indices were evaluated before the procedure. LAAV, LAAEF, LAAOA and LAA morphology were assessed via cardiac computed tomography, while LAAeV was evaluated by transesophageal echocardiography. All five LAA indices were calculated after averaging 3–5 cardiac cycles if patients were on sinus rhythm or after averaging 5–10 cardiac cycles if patients were on AF. Additional file 1: Tables S2 and S3 provides more details in ablation methodology, LAA imaging techniques, follow-up and antiarrhythmic medication protocols.

The study by Chang et al. [15] was included in LAAOA analysis, and the study by Tsao et al. [16] was included in LAAEF analysis, so as to avoid duplications. Similarly, the studies by Kim et al. [37] (2021; Int J Cardiovasc Imaging) and Kim et al. [39] (2021; JACC Clin Electrophysiol) were included in LAAV and LAAeV analyses, respectively, in order to avoid duplications. Finally, the study by Szegedi et al. [42] was included in LAA morphology analysis and data from the study by Simon et al. [41] were employed in LAAV, LAAeV and LAAOA analyses only.

As far as LAAeV analysis is concerned, Yoshida et al. [43] and Ma et al. [48] report data on PAF and Per-AF separately, while Kim et al. [39] reported data on early recurrence and early recurrence-free subgroups separately. Since these three studies did not report LAAeV for the whole study populations, we opted to make double entries for them.

### Clinical data

#### Left atrial appendage ejection fraction

**Fig. 2:**
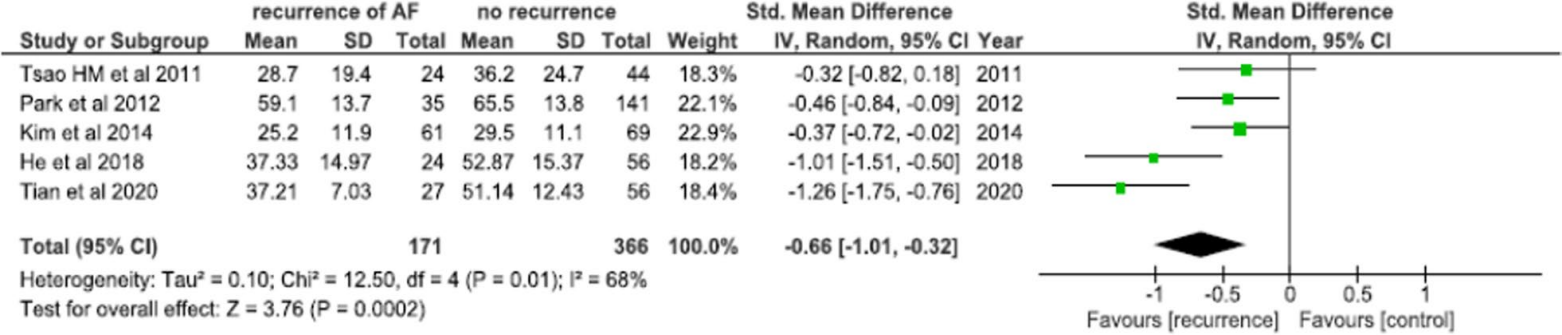
Mean difference in LAA ejection fraction between arrhythmia recurrence and arrhythmia free groups

A total of five studies involving 537 patients undergoing AFCA compared pre-ablation LAAEF between arrhythmia recurrence and arrhythmia free counterparts. LAAEF was significantly lower in patients with AF recurrence post-ablation [SMD = − 0.66; 95% CI (− 1.01, − 0.32); *I*^2^ = 68%; *p* = 0.0002] as compared to arrhythmia free controls (Fig. 2).

#### Left atrial appendage volume

**Fig. 3:**
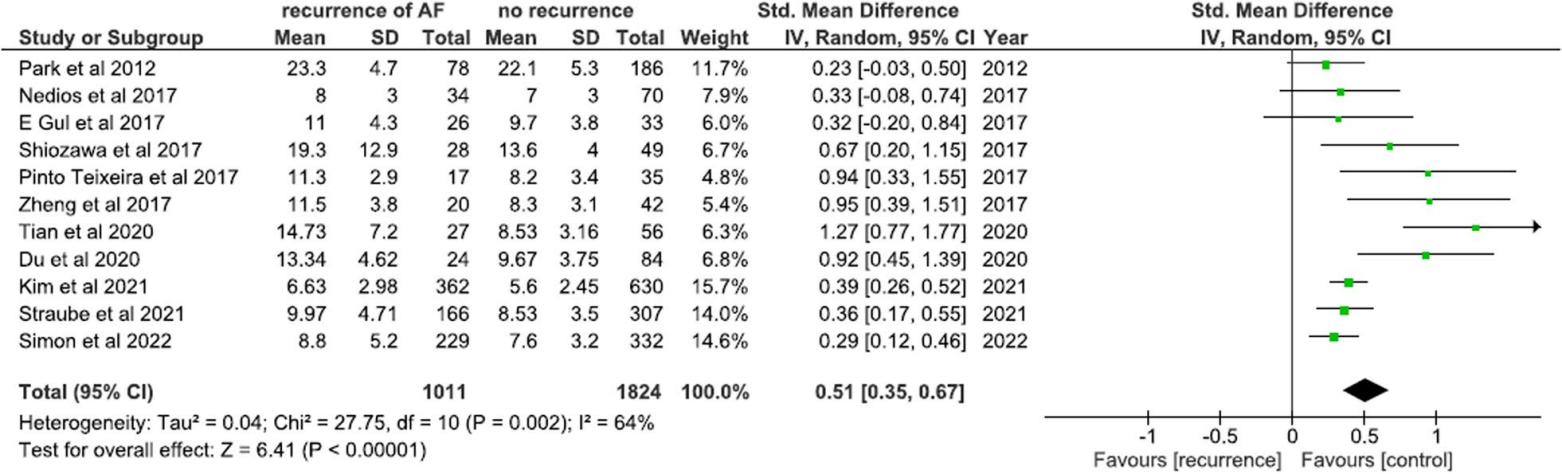
Mean difference in LAA volume between arrhythmia recurrence and arrhythmia free groups

A total of eleven studies involving 2835 patients undergoing AFCA compared pre-ablation LAAV between arrhythmia recurrence and arrhythmia free counterparts. LAAV was significantly higher in patients with AF recurrence post-ablation (SMD = 0.51; 95% CI 0.35–0.67; *I*^2^ = 64%; *p* < 0.00001) as compared to arrhythmia free controls (Fig. 3). We have also conducted a LAA volume subgroup analysis for pulmonary vein isolation (PVI) only and PVI plus additional lines subgroups and our findings remained robust (see Additional file 1: Fig. S1).

#### Left atrial appendage morphology

**Fig. 4:**
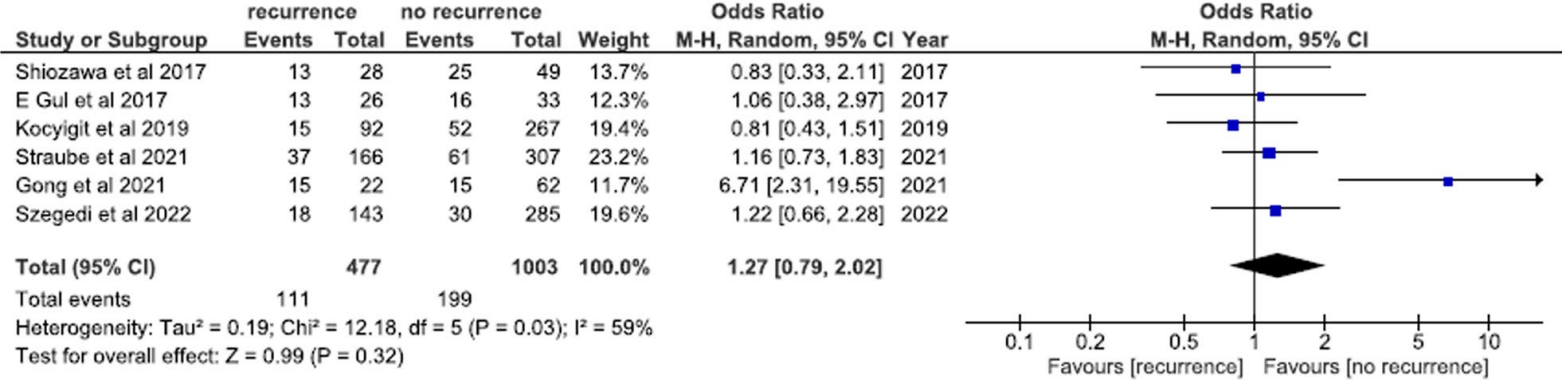
Chicken wing versus non-chicken wing LAA morphology between arrhythmia recurrence and arrhythmia free groups

A total of six studies involving 1480 patients undergoing AFCA compared pre-ablation chicken wing versus non-chicken wing LAA morphology between arrhythmia recurrence and arrhythmia free groups. LAA morphology was not predictive of AF recurrence post-ablation (chicken wing morphology; OR = 1.27; 95% CI 0.79–2.02; *I*^2^ = 59%; *p* = 0.32) (Fig. 4). A subgroup analysis for pulmonary PVI only and PVI plus additional lines subgroups did not reveal any arrhythmia recurrence difference between patients featuring chicken wing versus non-chicken wing LAA morphology (see Additional file 1: Fig. S2).

#### Left atrial appendage orifice area

**Fig. 5:**
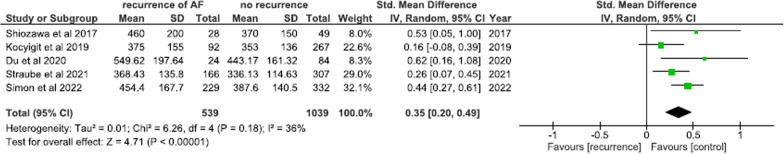
Mean difference in LAA orifice area between arrhythmia recurrence and arrhythmia free groups

A total of five studies involving 1578 patients undergoing AFCA compared pre-ablation LAAOA between arrhythmia recurrence and arrhythmia free counterparts. LAAOA was significantly higher in patients with AF recurrence post-ablation (SMD = 0.35; 95% CI 0.20–0.49; *I*^2^ = 36%; *p* < 0.00001) as compared to arrhythmia free controls (Fig. 5).

#### Left atrial appendage emptying velocity

**Fig. 6:**
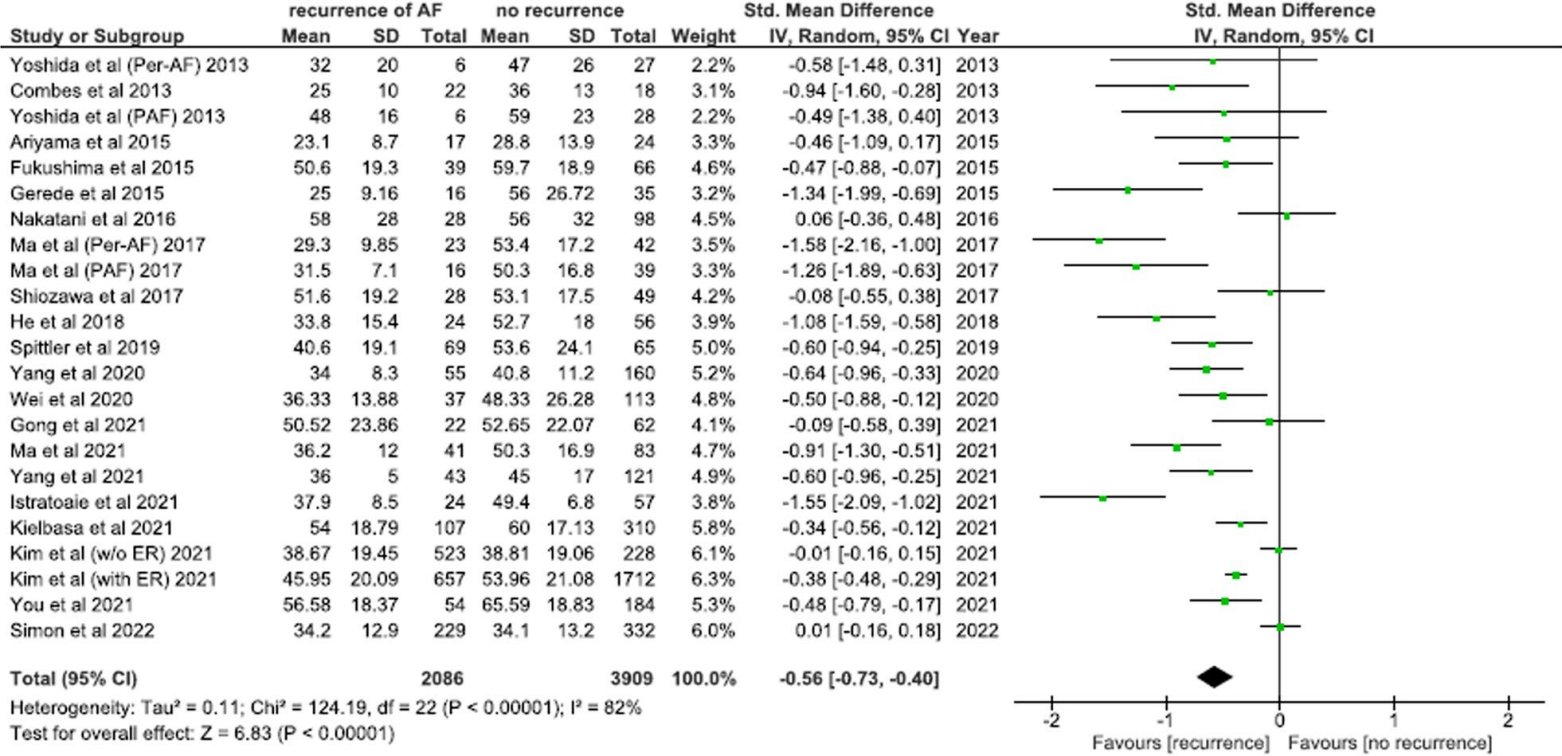
Mean difference in LAA emptying velocity between arrhythmia recurrence and arrhythmia free groups

A total of twenty studies involving 5995 patients undergoing AFCA compared pre-ablation LAAeV between arrhythmia recurrence and arrhythmia free counterparts. LAAeV was significantly reduced in patients with AF recurrence post-ablation [SMD = − 0.56; 95% CI − 0.73:− 0.40; *I*^2^ = 82%; *p* < 0.00001] as compared to arrhythmia free controls (Fig. 6). Two subgroup analyses were conducted and found that these observations remain robust for different ablation techniques [PVI only and PVI plus additional lines subgroups (see Additional file 1: Fig. S3)] and AF subtypes [paroxysmal and persistent AF subgroups (see Additional file 1: Fig. S4)].

### Sensitivity analyses

Compared to the main analysis, results remained unchanged after pooling the data using a fixed-effects model. Leave-one out sensitivity analysis by iteratively removing one study at a time did not drastically changed results concerning LAAeV, LAAV and LAAEF. As far as LAA morphology analysis is concerned, the study by Gong et al. [31] was found to be a significant source of statistical heterogeneity since removal of this study reduced heterogeneity to 0%, though LAA chicken wing morphology did not reach statistical significance (OR 1.04; 95% CI 0.78–1.39; *I*^2^ = 0%; *p* = 0.77). Lastly, when the study of Kocyigit et al. [24] was removed, heterogeneity in LAAOA analysis was also significantly reduced (SMD = 0.39; 95% CI 0.36–0.52; *I*^2^ = 9%; *p* < 0.00001) (Additional file 1: Table S4).

### Risk of bias assessment

The quality assessment scores of the NOS are shown in Additional file 1: Table S5. Seventeen studies included in the featured analyses were of high quality, while the remaining seventeen were of moderate quality.

### Assessment of publication bias

Funnel plot distributions of the featured analyses indicated absence of publication bias and small study effect for LAAV and LAAeV outcomes (see Additional file 1: Figs. S5 and S6). For outcomes with less than ten studies funnel plots were not assessed.

## Discussion

In our meta-analysis, we evaluated 34 published studies involving over 8000 patients and found that increased LAA volume and LAA orifice area, as well as decreased LAA ejection fraction and LAA emptying velocity are associated with arrhythmia recurrence in a mixed population of AF subtypes (paroxysmal and persistent) and AFCA modalities (radiofrequency ablation and cryoablation). LAA morphology (chicken wing versus non-chicken wing) was not associated with AF recurrence.

From a clinical perspective, the magnitude of the absolute difference in above-mentioned indices cannot be gauged by standardized mean difference employed in our analyses [49], yet we believe that our findings support the anatomic-mechanistic involvement of LAA in the myopathic AF substrate. In a recently published study, Vaishnav et al. found that unfavorable LA anatomy (including pulmonary vein ostial diameter and antral circumference) had a sensitivity and specificity above 80% to predict long-term arrhythmia recurrence post-cryoablation [50]. Furthermore, in a prospective sub-study of CryoLAEF we have shown that LAA function is improved after pulmonary vein isolation (radiofrequency ablation or cryoablation) in patients with paroxysmal AF [10].

As previously stated, the majority of the included studies employed a mixed population of paroxysmal and persistent AF subjects undergoing wide antral circumferential ablation with further ablation lines and substrate modification in most of the cases (see Table 1 and Additional file 1: Table  S4). This heterogeneous population and the lack of separately reported data prevented us from conducting some sub-analysis concerning AF subtype and ablation strategy. Yet, we found that both LAAeV and LAAV associations with arrhythmia recurrence remain robust for different ablation techniques (PVI only and PVI plus additional lines subgroups) and AF subtypes (paroxysmal and persistent AF subgroups). Of note, some of the available studies in the field report prediction statistics and support the validity of our findings (see Table 2).

**Table 2:** Arrhythmia recurrence prediction statistics in each individual study

Author/Year	LAA index	Arrhythmia recurrence prediction statistics	Significant	Adjustment variables
Park [27]	LAAV	HR(uni): 0.98 (95% CI 0.918–1.042)	No	N/A
Machino-Ohtsuka [38]	LAAeV	OR(adj): 1.76 (95% CI 0.94–3.29)	No	LAA wall velocity, mean of peak LA strain at systole
Kim 2014	LAAEF	LAAEF ≤ 20%; OR(uni):1.65 (95% CI 057–4.78)	No	N/A
Gerede [45]	LAAeV	LAAeV < 30 cm/s OR(adj): 1.129 (95% CI 1.115–1.228)	Yes (*p* = 0.004)	Presence of mitral annular calcification, LA diameter, pulmonary venous flow systolic wave velocity, and left atrial spontaneous echo contrast
Fukushima [46]	LAAeV	LAAeV < 48.5 cm/sec OR(adj): 2.68 (95% CI 1.136–6.318)	Yes (*p* = 0.024)	Age, gender, left atrial volume index, time from the onset of the P-wave of the surface electrocardiogram to the peak A’-wave of the atrial tissue Doppler tracing
Ma 2016	LAAeV	PAF; OR(adj): 0.88 (95% CI 0.79–0.98) Per-AF; OR(adj): 0.81 (95% CI 0.68–0.94)	Yes (*p* = 0.023)	Heart rate, systolic blood pressure, NT-proBNP ≥ 291 pg/ml
Zheng [19]	LAAV	HR(adj): 1.32 (95% CI 1.12–1.51)	Yes (*p* < 0.001)	AF type
Shiozawa [17]	LAAeV LAAV	HR (uni):1.00 (95% CI 0.98—1.02) HR (adj): 1.03 (95% CI 1.00–1.06)	No Yes (*p* = 0.04)	Use of statins, NT-proBNP
Pinto Teixeira [21]	LAAV	HR(adj): 1.32 (95% CI 1.12–1.55)	Yes (*p* < 0.001)	AF type
He [23]	LAAeV LAAEF	OR(adj): 0.934 (95% CI 0.975–0.997) OR(adj): 0.903 (95% CI 0.822–0.992)	Yes (*p* = 0. 042) Yes (*p* = 0. 033)	LA volume index, LA diameter, LAA minimum volume, LAA filling velocity
Kocyigit [24]	LAA_m	Non-CW; HR(adj): 0.731 (95% CI 0.403–1.328)	No	Age, AF type, LA diameter
Du [25]	LAAV LAAOA	HR(adj): 1.343(95% CI 1.114–1.619) HR (adj): 0.992(95% CI 0.983–1.001)	Yes (*p* = 0.002) No	AF type, LA diameter, LA volume, NT-proBNP, additional ablation lines
Tian [26]	LAAV LAAEF	HR(adj): 1.160 (95% CI 1.095–1.229) HR(adj): 0.790 (95% CI 0.657–0.950)	Yes (*p* = 0.000) Yes (*p* = 0.012)	CHA_2_DS_2_-VAS_C_ score, Heart Failure, LA ejection fraction, LA volume
Wei [29]	LAAeV	HR (uni):0.046 (95%CI 0.005–0.399)	Yes (*p* = 0.005)	N/A
Straube [30]	LAAV LAAOA LAA_m	HR(uni): 1.05 (95%CI 1.025–1.078) HR(uni): 1.20 (95% CI 1.100–1.300) CW; HR(uni): 1.13 (95% CI 0.781–1.624)	Yes (*p* < 0.0001) Yes (*p* = 0.001) No	N/A
Gong [31]	LAAeV LAA_m	OR(adj): 0.980 (95% CI 0.907–1.059) CW; OR(adj):8.13 (95% CI 1.94–34.02)	No Yes (*p* = 0.004)	Age, gender, AF type, ablation strategy, use of statins, LAA orifice diameter, LAA lobe number, LAA area
Yang 2021	LAAeV	LAAVeV < 0.37 m/s HR: 2.32 (95%CI 1.177–4.227)	Yes (*p* = 0.014)	AF duration, LA diameter, NT-proBNP, Heart Failure, LVEF, coronary heart disease
You [33]	LAAeV	OR( adj): 0.979 (95% CI 0.961–0.997)	Yes (*p* = 0.023)	LA dimensions
Istratoaie [34]	LAAeV	HR (uni):0.856 (95% CI 0.807–0.908)	Yes (*p* < 0.001)	N/A
Ma [35]	LAAeV	OR(adj): 0.940 (95% CI 0.896 − 0.987)	Yes (*p* = 0.011)	Age, NT-proBNP, AF type, LVEF, global longitudinal strain, left ventricular mass index, left ventricular volume index
Kielbasa 2021	LAAeV	LAAVeV < 45 cm/s HR(adj): 1.63 (95%CI 1.06–2.49)	Yes (*p* = 0.02)	LA dimensions, patent foramen ovale
Kim [37]	LAAV	LAAV index (≥ 7 ml/m2) HR (adj): 1.66 (95% CI 1.25–2.22)	Yes (*p* = 0.006)	LAV index, stroke, diabetes mellitus, obesity, heart failure, AF type, age
Kim [39]	LAAeV	HR (adj): 1.00 (95% CI 0.99- 1.00)	No	Age, AF type, heart failure, obesity, LA diameter, LVEF, CHA_2_DS_2_-VASc score, early arrhythmia recurrence
Spittler [40]	LAAeV	N/R	Yes (*p* = 0.026)	LAV index, arterial hypertension, AF duration, coronary heart disease, MR, SR at baseline, RAV index
Simon [41]	LAAeV LAAOA LAAV	PAF; HR (adj):1.01 (95% CI 1.00–1.02) Per-AF; HR (adj): 1.00 (95% CI 0.98–1.02) PAF; HR (adj): 1.00 (95% CI 1.00–1.00) Per-AF; HR(adj): 1.00 (95% CI 1.00–1.01) PAF; HR (adj): 1.00 (95% CI 0.93–1.06) Per-AF; HR(adj): 1.06 (95% CI 1.01–1.12)	No No No No No Yes (*p* = 0.029)	Age, gender, obesity, arterial hypertension, dyslipidemia, diabetes mellitus, stroke, coronary heart disease, thyroid disease, LVEF, LAV index, chronic kidney disease, LAAV, LAAeV, LAAOA, pre-ablation antiarrhythmic drug usage
Szegedi [42]	LAA_m	CW; HR (adj): 1.51 (95% CI 0.81–2.82)	No	Age, gender, obesity, arterial hypertension, dyslipidemia, diabetes mellitus, stroke, coronary heart disease, thyroid disease, LVEF, LAV index, chronic kidney disease, LAAV, LAAeV, LAAOA

*PAF* paroxysmal atrial fibrillation, *Per-AF* persistent atrial fibrillation, *LA* left atrium, *LAV* LA volume, *RAV* right atrium volume, *LAA* left atrial appendage, *LAAV* LAA volume, *LAAeV* LAA emptying velocity, *LAAOA* LAA orifice area, *LAAEF* LAA ejection fraction, *LAA_m* LAA morphology, chicken wing versus non-chicken wing, *HR(adj)* adjusted hazard ratio, *HR(uni)* univariate or unadjusted hazard ratio, *OR(adj)* adjusted odds ratio, *OR(uni)* univariate or unadjusted odds ratio, *95% CI* 95% confidence intervals, *CW* chicken wing morphology of LAA, *N/A* not applicable, *N/R* not reported, *NT-proBNP* N-terminal pro-brain natriuretic peptide, *LVEF* left ventricular ejection fraction, *MR* mitral regurgitation, *SR* sinus rhythm

Specifically, Kim et al. [37] utilized LAA volume index data and found that LAA volume index ≥ 7 ml/m^2^ is associated with 66% increased risk of AF recurrence. Importantly, this association remained significant after adjustment for clinically relevant predictors of arrhythmia recurrence such age, LA volume index, diabetes mellitus, heart failure, body mass index and AF subtype [9, 51, 52]. Tian et al. have also found that increased LAA volume increases AF recurrence risk by 16%, and this finding is independent of LA volume and CHA_2_DS_2_-VASc score [26]. Similarly, Du et al. suggested that LAA volume increases arrhythmia recurrence risk by 34% after adjustment for LA volume, AF subtype and additional ablation lines [25].

As far as LAA ejection fraction is concerned, the studies by He et al. and Tian et al. underscore that increased LAA ejection fraction is associated with a reduction in AF recurrence ranging from 10 to 20% after adjustment for LA volume [23, 26]. Regarding LAA orifice area, both Du et al. and Simon et al. have adjusted their finding for LA volume and found that LAA orifice area is not an independent predictor of arrhythmia recurrence [25, 41].

Lastly, many investigators have examined LAA emptying velocity as an independent predictor of arrhythmia recurrence post-AFCA (see Table 2) and the vast majority of them found a significant association. Both Fukushima et al. and He et al. have adjusted for LA volume index and showed that reduced LAA emptying velocity is an independent predictor of AF recurrence [23, 46]. In addition, Fukushima et al. suggested that a LAA emptying velocity below 48.5 cm/sec is associated with a nearly threefold increase in AF recurrence risk [46]. On the contrary, Simon et al. have adjusted for LA volume index, among other variables, and found that LAA emptying velocity is not predictive of AF recurrence post-ablation in both paroxysmal and persistent AF subtypes [41].

In view of our findings it is evident that LAA is affected by the underlying AF related atrial cardiomyopathy, yet in a varying degree. While it is highly unlikely that future studies would prove that LAA indices can single-out ideal AFCA candidates, it should be kept in mind that LAA morphofunctional features could be an incremental and valuable addition in artificial intelligence algorithms and AF recurrence prediction scores.

Of note, machine learning models are proven to outperform existing arrhythmia recurrence scores such as APPLE and CHA_2_DS_2_-VASc and when combining clinical, electrocardiographic and electrophysiology data they achieve an area under the receiver operating characteristics curve (AUC) of 0.86 [53]. Interestingly, a recently published study by Zhou et al. suggested that a deep learning algorithm utilizing four variables (LA volume, LAA volume, N-terminal pro-BNP levels and AF subtype) has superior performance than conventional statistical analysis in AF recurrence prediction and an average C-index of 0.76 [54]. While methodologically different from our analysis, another novel artificial intelligence approach found that a deep learning algorithm analyzing post-ablation (within 24 h) LA indices (diameter, emptying fraction and strain rate) outperforms conventional logistic regression models in predicting late AF recurrence [55].

Since cardiac computed tomography (CCT) and transesophageal echocardiography are common and readily accessible pre-operative imaging modalities in AFCA candidates, automated LAA anatomic and functional indices estimations could be rapidly, easily and timely integrated in clinical decision making to facilitate better long term outcomes. For the time being, deep learning techniques can accurately estimate LA volume and geometry via CCT data and assist in arrhythmia recurrence prediction post-AFCA [56, 57]. Lastly, cardiac magnetic resonance imaging datasets have been utilized in a proof-of-concept study showing that deep learning can assist in patient specific ablation strategy selection [58].

Whether implementation of LAA datasets in artificial intelligence approaches could facilitate arrhythmia recurrence prediction, ablation strategy selection or even duration of antiarrhythmic drugs post-ablation remains to be proven. Importantly, artificial intelligence models for clinical decision making should be further tested since ethical, legal and social considerations remain both relevant and hotly debated [59–62].

### Limitations

The present meta-analysis should be interpreted taking into account its limitations. First, this is a study-level meta-analysis providing average treatment effects. The lack of patient-level data prevented us from assessing the impact of baseline clinical and procedural characteristics on treatment effects. Yet, some crucial subgroup analyses (see Additional file 1: Figs. S1–S4) were conducted and showed that our findings remain robust for paroxysmal and persistent AF subgroups as well as different ablation approaches (PVI with or without additional ablation lines). Second, results of this study were grounded on a relatively small number of case–control studies of moderate quality. In particular, comparability between groups may be inadequate, since adjustment for confounding factors was not part of the initial design in the majority of the studies. Finally, moderate statistical heterogeneity was observed, which can imply methodology issues, such as different cardiac imaging and catheter ablation modalities and techniques, diverse population characteristics and post-ablation AAD protocols (see Additional file 1: Table S2 and S3). As previously stated, overall LAA indices calculation, follow-up and AAD administration were largely comparable in the included studies. In addition, considering that no strong evidence exist on post-AFCA AAD administration, follow-up protocol and additional ablation lesions beyond pulmonary vein isolation in AF subjects [2, 63], any heterogeneity of the included studies is indicative of the real-word practice.

## Conclusions

LAA morphofunctional indices differ between patients suffering from arrhythmia recurrence post-ablation and arrhythmia free counterparts. Their clinical significance and their role in better risk stratification of catheter ablation AF candidates is an open research question that definitely merits further investigation.

## Supplementary Information

**Additional file 1.**

## Data Availability

Dataset available upon request.

## Abbreviations

AAD: Antiarrhythmic drugs
AF: Atrial fibrillation
AFCA: Atrial fibrillation catheter ablation
AUC: Area under the receiver operating characteristics curve
CI: Confidence intervals
CCT: Cardiac computed tomography
LA: Left atrium
LAA: Left atrial appendage
LAAV: LAA volume
LAAEF: LAA ejection fraction
LAAeV: LAA emptying velocity
LAAOA: LAA orifice area
NOS: Newcastle–Ottawa scale
SMD: Standardized mean difference

## References

[1] Benjamin EJ, Muntner P, Alonso A (2019). Heart disease and stroke statistics-2019 update: a report from the American heart association. Circulation.

[2] Hindricks G, Potpara T, Dagres N (2021). 2020 ESC guidelines for the diagnosis and management of atrial fibrillation developed in collaboration with the European association for cardio-thoracic surgery (EACTS). Eur Heart J.

[3] Guo Y, Lane DA, Wang L (2020). Mobile health technology to improve care for patients with atrial fibrillation. J Am Coll Cardiol.

[4] Wazni OM, Dandamudi G, Sood N (2021). Cryoballoon ablation as initial therapy for atrial fibrillation. N Engl J Med.

[5] Andrade JG, Wells GA, Deyell MW (2021). Cryoablation or drug therapy for initial treatment of atrial fibrillation. N Engl J Med.

[6] Perino AC, Leef GC, Cluckey A (2019). Secular trends in success rate of catheter ablation for atrial fibrillation: the SMASH-AF cohort. Am Heart J.

[7] Dretzke J, Chuchu N, Agarwal R (2020). Predicting recurrent atrial fibrillation after catheter ablation: a systematic review of prognostic models. EP Eur.

[8] Delgado V, Di Biase L, Leung M (2017). Structure and function of the left atrium and left atrial appendage. J Am Coll Cardiol.

[9] Njoku A, Kannabhiran M, Arora R (2018). Left atrial volume predicts atrial fibrillation recurrence after radiofrequency ablation: a meta-analysis. EP Eur.

[10] Giannopoulos G, Kekeris V, Vrachatis D (2018). Effect of pulmonary vein isolation on left atrial appendage flow in paroxysmal atrial fibrillation. Pacing Clin Electrophysiol.

[11] Liberati A, Altman DG, Tetzlaff J (2009). The PRISMA statement for reporting systematic reviews and meta-analyses of studies that evaluate healthcare interventions: explanation and elaboration. BMJ.

[12] Wells, G. A, Shea, B., O’Connel D et al. (2009) The Newcastle-Ottawa scale (NOS) for assessing the quailty of nonrandomised studies in meta-analyses. http://www.ohri.ca/programs/clinical_epidemiology/oxfordhtm2009Feb1. 2009

[13] Wan X, Wang W, Liu J, Tong T (2014). Estimating the sample mean and standard deviation from the sample size, median, range and/or interquartile range. BMC Med Res Methodol.

[14] Stroup DF, Berlin JA, Morton SC, Olkin I, Williamson GD, Rennie D, Moher D, Becker BJ, Sipe TATS (2000). Meta-analysis of observational studies in epidemiology: a proposal for reporting. Meta-analysis Of Observational Studies in Epidemiology (MOOSE) group. JAMA.

[15] Chang S-H, Tsao H-M, Wu M-H (2007). Morphological changes of the left atrial appendage after catheter ablation of atrial fibrillation. J Cardiovasc Electrophysiol.

[16] Tsao H-M, Hu W-C, Wu M-H (2011). Quantitative analysis of quantity and distribution of epicardial adipose tissue surrounding the left atrium in patients with atrial fibrillation and effect of recurrence after ablation. Am J Cardiol.

[17] Shiozawa T, Shimada K, Sekita G (2017). Left atrial appendage volume and plasma docosahexaenoic acid levels are associated with atrial fibrillation recurrence after catheter ablation. Cardiol Res.

[18] E Gul E, Boles U, Haseeb S (2017). Left Atrial Appendage characteristics in patients with Persistent Atrial Fibrillation undergoing catheter ablation (LAAPAF Study). J Atr Fibrillation.

[19] Zheng GA, Lin CY, Weng L, Chen JD (2017). Left atrial appendage volume is a valuable predictor of atrial fibrillation recurrence after radiofrequency catheter ablation. Zhonghua Xin Xue Guan Bing Za Zhi.

[20] Nakatani Y, Sakamoto T, Mizumaki K (2016). Coefficient of variation of P-wave duration is a novel atrial heterogeneity index to predict recurrence of atrial fibrillation after catheter ablation. J Cardiovasc Electrophysiol.

[21] Pinto Teixeira P, Martins Oliveira M, Ramos R (2017). Left atrial appendage volume as a new predictor of atrial fibrillation recurrence after catheter ablation. J Interv Card Electrophysiol.

[22] Nedios S, Koutalas E, Sommer P (2017). Asymmetrical left atrial remodelling in atrial fibrillation: relation with diastolic dysfunction and long-term ablation outcomes. Europace.

[23] He Y, Zhang B, Zhu F (2018). Transesophageal echocardiography measures left atrial appendage volume and function and predicts recurrence of paroxysmal atrial fibrillation after radiofrequency catheter ablation. Echocardiography.

[24] Kocyigit D, Yalcin MU, Gurses KM (2019). Impact of anatomical features of the left atrial appendage on outcomes after cryoablation for atrial fibrillation. J Cardiovasc Comput Tomogr.

[25] Du W, Dai M, Wang M (2020). Large left atrial appendage predicts the ablation outcome in hypertensive patients with atrial fibrillation. J Electrocardiol.

[26] Tian X, Zhang X-J, Yuan Y-F (2020). Morphological and functional parameters of left atrial appendage play a greater role in atrial fibrillation relapse after radiofrequency ablation. Sci Rep.

[27] Park H-C, Shin J, Ban J-E (2013). Left atrial appendage: morphology and function in patients with paroxysmal and persistent atrial fibrillation. Int J Cardiovasc Imaging.

[28] Yang Z, Xu M, Zhang C (2021). A predictive model using left atrial function and B-type natriuretic peptide level in predicting the recurrence of early persistent atrial fibrillation after radiofrequency ablation. Clin Cardiol.

[29] Wei Y, Liu S, Yu H (2020). The predictive value of growth differentiation factor-15 in recurrence of atrial fibrillation after catheter ablation. Mediators Inflamm.

[30] Straube F, Pongratz J, Hartl S (2021). Cardiac computed tomography angiography-derived analysis of left atrial appendage morphology and left atrial dimensions for the prediction of atrial fibrillation recurrence after pulmonary vein isolation. Clin Cardiol.

[31] Gong S, Zhou J, Li B (2021). The association of left atrial appendage morphology to atrial fibrillation recurrence after radiofrequency ablation. Front Cardiovasc Med.

[32] Yang W, Zhao Q, Yao M (2021). The prognostic significance of left atrial appendage peak flow velocity in the recurrence of persistent atrial fibrillation following first radiofrequency catheter ablation. J Thorac Dis.

[33] You L, Zhang X, Yang J (2021). The long-term results of three catheter ablation methods in patients with paroxysmal atrial fibrillation: a 4-year follow-up study. Front Cardiovasc Med.

[34] Istratoaie S, Vesa Ștefan C, Cismaru G (2021). Value of left atrial appendage function measured by transesophageal echocardiography for prediction of atrial fibrillation recurrence after radiofrequency catheter ablation. Diagnostics.

[35] Ma X-X, Wang A, Lin K (2021). Incremental predictive value of left atrial strain and left atrial appendage function in rhythm outcome of non-valvular atrial fibrillation patients after catheter ablation. Open Hear.

[36] Kiełbasa G, Bednarek A, Bednarski A (2021). Patent foramen ovale and left atrial appendage flow velocity predict atrial fibrillation recurrence post cryoballoon ablation. Kardiol Pol.

[37] Kim DY, Kim YG, Choi J-I (2021). A novel predictive model for late recurrence after catheter ablation for atrial fibrillation using left appendage volume measured by cardiac computed tomography. Int J Cardiovasc Imaging.

[38] Machino-Ohtsuka T, Seo Y, Ishizu T (2013). Significant improvement of left atrial and left atrial appendage function after catheter ablation for persistent atrial fibrillation. Circ J.

[39] Kim YG, Boo KY, Choi J-I (2021). Early recurrence is reliable predictor of late recurrence after radiofrequency catheter ablation of atrial fibrillation. JACC Clin Electrophysiol.

[40] Spittler R, Bahlke F, Hoffmann BA (2019). Predictors of successful complex catheter ablation for persistent atrial fibrillation despite failure of targeted procedural arrhythmia termination. J Cardiovasc Electrophysiol.

[41] Simon J, El Mahdiui M, Smit JM (2022). Left atrial appendage size is a marker of atrial fibrillation recurrence after radiofrequency catheter ablation in patients with persistent atrial fibrillation. Clin Cardiol.

[42] Szegedi N, Simon J, Szilveszter B (2022). Abutting left atrial appendage and left superior pulmonary vein predicts recurrence of atrial fibrillation after point-by-point pulmonary vein isolation. Front Cardiovasc Med.

[43] Yoshida N, Okamoto M, Hirao H (2013). Efficacy of pulmonary vein isolation on left atrial function in paroxysmal and persistent atrial fibrillation and the dependency on its baseline function. Echocardiography.

[44] Combes S, Jacob S, Combes N (2013). Predicting favourable outcomes in the setting of radiofrequency catheter ablation of long-standing persistent atrial fibrillation: a pilot study assessing the value of left atrial appendage peak flow velocity. Arch Cardiovasc Dis.

[45] Gerede DM, Candemir B, Vurgun VK (2015). Prediction of recurrence after cryoballoon ablation therapy in patients with paroxysmal atrial fibrillation. Anatol J Cardiol.

[46] Fukushima K, Fukushima N, Ejima K (2015). Left atrial appendage flow velocity and time from P-wave onset to tissue Doppler-derived A’ predict atrial fibrillation recurrence after radiofrequency catheter ablation. Echocardiography.

[47] Ariyama M, Kato R, Matsumura M (2015). Left atrial appendage wall-motion velocity associates with recurrence of nonparoxysmal atrial fibrillation after catheter ablation. Echocardiography.

[48] Ma X-X, Zhang Y-L, Hu B (2017). Association between left atrial appendage emptying velocity, N-terminal plasma brain natriuretic peptide levels, and recurrence of atrial fibrillation after catheter ablation. J Interv Card Electrophysiol.

[49] Takeshima N, Sozu T, Tajika A (2014). Which is more generalizable, powerful and interpretable in meta-analyses, mean difference or standardized mean difference?. BMC Med Res Methodol.

[50] Vaishnav AS, Alderwish E, Coleman KM (2020). Anatomic predictors of recurrence after cryoablation for atrial fibrillation: a computed tomography based composite score. J Interv Card Electrophysiol.

[51] D’Ascenzo F, Corleto A, Biondi-Zoccai G (2013). Which are the most reliable predictors of recurrence of atrial fibrillation after transcatheter ablation?: A meta-analysis. Int J Cardiol.

[52] Anselmino M, Matta M, D’Ascenzo F (2015). Catheter ablation of atrial fibrillation in patients with diabetes mellitus: a systematic review and meta-analysis. Europace.

[53] Tang S, Razeghi O, Kapoor R (2022). Machine learning-enabled multimodal fusion of intra-atrial and body surface signals in prediction of atrial fibrillation ablation outcomes. Circ Arrhythmia Electrophysiol.

[54] Zhou X, Nakamura K, Sahara N (2022). Deep learning-based recurrence prediction of atrial fibrillation after catheter ablation. Circ J.

[55] Hwang Y-T, Lee H-L, Lu C-H (2021). A novel approach for predicting atrial fibrillation recurrence after ablation using deep convolutional neural networks by assessing left atrial curved M-mode speckle-tracking images. Front Cardiovasc Med.

[56] Abdulkareem M, Brahier MS, Zou F (2022). Generalizable framework for atrial volume estimation for cardiac CT images using deep learning with quality control assessment. Front Cardiovasc Med.

[57] Chen H-H, Liu C-M, Chang S-L (2020). Automated extraction of left atrial volumes from two-dimensional computer tomography images using a deep learning technique. Int J Cardiol.

[58] Muffoletto M, Qureshi A, Zeidan A (2021). Toward patient-specific prediction of ablation strategies for atrial fibrillation using deep learning. Front Physiol.

[59] Rubeis G (2022). iHealth: The ethics of artificial intelligence and big data in mental healthcare. Internet Interv.

[60] Lorenzini G, Arbelaez Ossa L, Shaw DM, Elger BS (2023). Artificial intelligence and the doctor-patient relationship expanding the paradigm of shared decision making. Bioethics.

[61] d’Elia A, Gabbay M, Rodgers S (2022). Artificial intelligence and health inequities in primary care: a systematic scoping review and framework. Fam Med Commun Heal.

[62] Rajkomar A, Hardt M, Howell MD (2018). Ensuring fairness in machine learning to advance health equity. Ann Intern Med.

[63] Calkins H, Hindricks G, Cappato R (2018). 2017 HRS/EHRA/ECAS/APHRS/SOLAECE expert consensus statement on catheter and surgical ablation of atrial fibrillation. Europace.

