# Supplementary material for: Left atrial appendage morphofunctional indices could be predictive of arrhythmia recurrence post-atrial fibrillation ablation: a meta-analysis
Source: Egypt Heart J. 2023 Apr 20;75:29. doi: 10.1186/s43044-023-00356-3 (PMC10119349; doi:10.1186/s43044-023-00356-3)

**Supplementary Table 1**: Search Strings

| **Database** | **Keywords/Search String** | **Filters** | **Results** |
| --- | --- | --- | --- |
| **PubMed** | (atrial appendage) AND (atrial fibrillation ablation recurrence) | none | 329 |
| **Cochrane Library** | atrial appendage and atrial fibrillation recurrence | MeSH | 59 |
| **medRxiv** | atrial appendage | None | 70 |
| **ClinicalTrials.gov** | Completed Studies \| Studies With Results \| atrial appendage | completed studies, with results | 12 |

**Supplementary Table 2**: Additional data on imaging techniques, follow-up and anti-arrhythmic medication protocols.

| **Author/**  **Year** | **Imaging Modality** | **CT technique/manufacturer** | **CA technique/manufacturer** | **TEE manufacturer** | **FU protocol** | **AAD protocol** |
| --- | --- | --- | --- | --- | --- | --- |
| Tsao 2011 | CCT | 64-slice multidetector computed tomographic scanner  (Aquilion 64 CFX, Toshiba Medical System, Tokyo, Japan) | NavX system, 4-mm-tip ablation catheter (EP Technologies, Boston Scientific, Inc., Natick, Massachusetts) | N/A | 24h Holter or 1 week cardiac event recording at 2 weeks & q 1-3 mo thereafter | 2 mo post-AFCA |
| Park 2012 | CCT | 3-dimensional multi-slice helical contrast-CT | NavX system (St Jude Medical, St Paul,MN,USA) or CARTO system (Biosense Webster, Diamond Bar, CA, USA) | N/A | 24-48h Holter, ECG at 3, 6, 9, 12, 18, 24, 30 & 36 mo | N/R |
| Machino-Ohtsuka  2013 | TEE | N/A | 7-Fr decapolar ring catheter (Lasso, Biosense Webster, Diamond Bar, CA, USA)  7.5 Fr irrigated catheter with a 3.5 mm distal electrode (ThermoCool, Biosense, Webster) | X7-2t TEE transducer (Philips iE33, Philips Medical Systems, Andover, MA, USA) | 24-48h Holter, ECG at 2 weeks, 1, 3, 6, 12 mo | Patients with AF recurrence were treated temporarily with AAD |
| Yoshida 2013 | TEE | N/A | CARTO, irrigation catheter (Thermocool, Biosense Webster, point-by-point ablation) | Vivid 7 ultrasound system (General Electric Healthcare, Milwaukee, WI, USA) and iE33 (Philips Medical Systems, Bothell,WA, USA) | N/R | 6 mo post-AFCA (if already on AAD) |
| Combes 2013 | TEE | N/A | 4-mm externally irrigated-tip ablation catheter (Thermocool, Biosense Webster, Carto 3; Biosense Webster) | 7-MHz transducer (Vividi; General Electric Medical Health, Horten, Norway) | TEE, ECG& 24h Holter at 3,6,12 mo | 3 mo amiodarone (if already on AAD) |
| Kim 2014 | TEE | N/A | NavX™ (St. Jude Medical, St, Paul, MN, USA) or  CARTO™ (Biosense Webster, Diamond Bar, CA, USA)  20-pole Lasso deflectable mapping catheter( Biosense Webster, 3.5 mm irrigated tip catheter (NaviStar ThermoCool, 7.5 Fr., D-curve, Biosense, Webster) | iE33 (Philips Medical Systems, Andover, MA, USA) | ECG, 24h Holter at 2 weeks, q 2-3 months thereafter | 3mo post-AFCA |
| Gerede 2015 | TEE | N/A | 28-mm cryoballoon catheter (Arctic Front ©; Medtronic CryoCath LP, Kirkland, Canada) | 5 MHz biplane phased array transducer (Vivid S5; GE, Horten, Norway) | 12-lead ECG, standard TTE, 24h Holter at 3, 6, and 12 mo | 3mo post-AFCA |
| Fukushima 2015 | TEE | N/A | 3.5-mm cooled-tip catheter (Navistar ThermoCool; Biosense Webster Inc., Diamond Bar, CA, USA) | Philips Healthcare Bothell, WA, USA) with a broadband S4 transducer (2–4 MHz) and an X7-2t TEE transducer | ECG, 24h Holter at 1, 2, 3, 6, 9, 12 mo post-AFCA, and then q 6 mo | up to 2 mo  (type 1 AAD) |
| Ariyama 2015 | TEE | N/A | saline-irrigated ablation catheter (Navistar Thermocool; Biosense Webster, Inc., Diamond Bar, CA, USA) | 5 MHz Probe (UST-5293S; Aloka) Vivid-7 system (General Electric Vingmed, Milwaukee, WI, USA) or Prosound alpha 10 system (Hitachi Aloka, Tokyo, Japan) | ECG ±24h Holter q 1–3 mo post AFCA | 3mo AAD  post-AFCA |
| Ma 2016 | TEE | N/A | N/R | iE33 (Philips Medical Systems, Koninklijke, Netherlands) | ECG,24h Holter at 3, 6, and 12 mo | N/R |
| Nakatani 2016 | TEE | N/A | NavX system (NavX with CFE software, St. Jude Medical Inc., St. Paul, MN, USA)  3.5-mm irrigated tip radiofrequency catheter (Safire, St. Jude Medical Inc.) | N/R | monthly visits to outpatient clinic w/ 24h Holter monitoring as needed | 3 mo post-AFCA |
| E Gul 2017 | CCT | 64-MDCT scanner or 320-Toshiba  (GE Healthcare, USA) | CF-sensed mapping and ablation catheter (TacticathTM Quartz, St. Jude Medical, St. Paul, MN, USA)  EnSite Velocity system (St. Jude Medical, St. Paul, USA) | N/A | 24h ECG Holter monitoring at 3, 6 mo, and yearly thereafter | N/R |
| Zheng 2017 | CCT | NA | CARTO system | N/A | 24h Holter monitoring at 3, 6 and 12 mo post-AFCA | N/R |
| Shiozawa 2017 | CCT | Aquilion ONE ViSION Edition™ (320-multidetector CT, Toshiba Medical Systems Corporation, Otawara, Japan) | open, irrigated tip (Thermocool Smarttouch; Biosense Webster), CARTO mapping system (Biosense Webster, Diamond Bar, CA, USA) | N/R | ECG, 24 h Holter monitoring at 1, 3, 6, and 12 mo | Patients with AF recurrence were treated temporarily with AAD |
| Pinto Teixeira 2017 | CCT | 64-detector scanner  (VCT LightSpeed, GE Healthcare) | circumferential pulmonary vein isolation using radiofrequency energy delivered by catheter with an open, irrigated tip | N/A | 12-lead ECG and 24 h Holter at 3, 6, 12 and 24 mo | 6mo post-AFCA (if already on AAD) |
| Nedios 2017 | CCT | multidetector 64- row helical system (Brilliance 64, Philips Medical Systems,Best, Netherlands) | EnSite™NavX™ (St. Jude Medical) or CARTO™ (Biosense Webster, Diamond Bar, CA, USA) | N/A | 7d Holter at 6, 12, 24, and 36 mo | AAD discontinuation |
| He 2018 | TEE | N/A | N/R | Philips EPIQ7C scanner  with an X7-2t 3D  (7–15 MHz) probe and a S5-1 (2.5– 5.0 MHz) 2D probe Qlab.10.5 (Philips Medical Systems, Andover, MA, USA) | ECG 24 h Holter at 3, 6, and 12 mo post- RFCA | N/R |
| Kocyigit 2019 | CCT | first-generation dual-source 64-slice multidetector CT scanner  (Somatom Definition; Siemens, Erlangen, Germany) | second or third-generation 28-mm CB catheter  (Arctic Front Advance™ and Arctic Front Advance ™ ST, Medtronic Inc.,Minneapolis, MN, USA) | N/A | ECG & 24 h Holter at 1, 3, 6 and 12 mo and then q 6 mo | 3mo post-AFCA |
| Du 2020 | CCT, TEE | PHILIPS Brilliance iCT 256 slice CT scanner  (Philips Medical Systems, MA, USA) | 3.5 mm irrigated  -tip catheter (Thermocool Smart TouchTM catheter, Biosense Webster, Diamond Bar, CA, USA)  circular catheter (Lasso, Biosense Webster, Diamond Bar, CA, USA)  3D electroanatomical mapping system  (CARTO-3, Biosense Webster, Diamond Bar, CA, USA) | Philips medical ultrasonography (IE 55 or SONOS 5500, Philips Medical Systems, MA,  USA) | ECG, 24h Holter at 3,6, 12 mo | 6 mo post-AFCA |
| Tian 2020 | CCT | Philips 256-slice spiral CT scanning | Carto 3 (Biosense Webster) circumferential pulmonary vein ablation (Lasso NAV eco, Biosense Webster), 3.5-mm irrigated tip ablation catheter (SmartTouch, Biosense Webster) | N/A | ECG or dynamic electrocardiogram at 3, 6 and 12 mo | N/R |
| Yang 2020 | TEE | N/A | CARTO system  catheter (3.5 mm, Smart Touch Catheter,Biosense Webster Inc., Diamond Bar, CA, USA) with thermocouple needle | Philips Epiq 7C heart ultrasound system (Netherlands) with a cardiac probe (X5-1, X7-2t) | ECG q mo, and a 24h Holter examination at least once a month | 3 mo (amiodarone 200 mg OD; propafenone 150 mg TID;  morerazine 150 mg TID;  sotalol 80 mg BID) |
| Wei 2020 | TEE | N/A | 3-D mapping CARTO 3 system, irrigated RF Smart Touch force-sensing catheter  (Biosense Webster, USA) | Vivid E9 equipped with a multiplane TEE probe | 12-lead ECG and 72 h Holter at 1, 3, 6, 12, 18, and 24 mo | 3mo amiodarone |
| Straube 2021 | CCT | 64-slice CT scanner  (Brilliance 64, Philips Medical Systems, Cleveland, OH, USA) | second-generation cryoballoon  (Arctic Front Advance™, Medtronic Inc., MN, USA) | N/A | 24 h Holter at 1, 3, 6, 12 mo | N/R |
| Gong 2021 | TEE | N/A | CARTO (Biosense Webster, Irvine, CA, USA)  CF ablation catheter (CARTO, Thermocool, Biosense Webster) | Philips IE33 color ultrasound with multi-planar 3D transesophageal probe X7-2t | 12-lead ECGs or 24h Holter monitoring at 3, 6, and 12 mo | Per-AF: 3mo propafenone or amiodarone |
| Yang 2021 | TEE | N/A | CARTO 3 system (Biosense Webster, USA) | IE Elite ultrasound | 24h Holter at 3,6,9, 12 mo and q 6 mo thereafter | 3mo amiodarone |
| You 2021 | TEE | N/A | *RF*: Carto 3, Biosense Webster, Lasso NAV eco, Biosense Webster,  3.5-mm irrigated tip ablation catheter (SmartTouch, Biosense Webster) *sg-CBA:* Arctic Front Advance Cardiac CryoAblation Catheter, ( Medtronic, Minneapolis, MN) *RF+ CB:* EnSite NavX 3D mapping system | iE33 machine equipped with X3-1 and X7-2t (Philips Medical Systems, Eindhoven, the Netherlands) | ECGs and 24h Holter weekly in the first month and then at 2, 3, 6, 9, and 12 mo | N/R |
| Istratoaie 2021 | TEE | N/A | Ensite NAVX Velocity (Saint-Jude Medical, Saint Paul, MN, USA) or a CARTO 3 system (Biosense Webster, Diamond Bar, CA, USA), open-irrigated 7 french 3.5 mm ablation catheter (Navistar Thermocol and Thermocool Smarttouch, Biosense Webster, Diamond Bar, CA, USA) or the FlexAbility irrigated ablation catheter (Saint-Jude Medical, Saint Paul, MN, USA) | Philips Affiniti50  (Philips Healthcare, Best, The Netherlands) with a 2–4 MHz microconvex transducer for ETT and a 7 MHz transducer for TEE | ECG and 24 h Holter recording at 3, 6, and 12 mo | N/R |
| Ma 2021 | TEE | N/A | CARTO mapping system (Biosense Webster, Irvine, California, USA) | iE33 ultrasound machines (Philips Medical Systems, Eindhoven, Netherlands) and EPIQ 7C (Philips Healthcare,Eindhoven, Netherlands) | 12-lead ECG and 24h Holter monitoring at 3, 6 and 12 mo | 3mo AAD |
| Kielbasa 2021 | TEE | N/A | sg-CBA Arctic Front Advance (Medtronic Inc., Minneapolis, MN, USA) | Vivid 9  (GE Healthcare, Horten, Norway) device with a 4D TEE probe | ECG, 24-72h Holter monitoring, or intracardiac electrogram from the implanted device at 3 and 6–9 mo and then advised once a year | discontinuation within 3mo |
| Kim 2021 | CCT | 64-detector scanner | EnSite NavX/Velocity  (St. Jude Medical, St. Paul, MN) or CARTO | N/A | ECG , 24h Holter at 3, 6, 9, and 12 mo | class Ic or III 3mo post-AFCA |
| Kim 2021 | TEE | N/A | EnSite NavX/Velocity (St. Jude Medical, St. Paul, MN) or CARTO | N/R | ECG , 24h Holter at 3, 6, 9, and 12 mo | class Ic or III 3mo post-AFCA |
| Spittler 2021 | TEE | N/A | NavX Ensite System (St. Jude Inc), irrigated‐tip ablation catheter with a 3.5mm tip electrode (ThermoCool SmartTouch SF Uni‐Directional Navigation Catheter; 7 French; Biosense Webster Inc) | CX50 or iE33 (Philips Inc., Bothell, WA, US) | 48h Holter, ECG at 3,6,12 mo | N/R |
| Simon 2022 | CCT,TEE | 256-slice scanner  (Brilliance iCT 256, Philips Healthcare, Best, The Netherlands) | CARTO (Biosense Webster, Inc., Diamond Bar, CA, USA) or ENSITE (St. Jude Medical, Inc., MN, USA), non-contact force-sensing catheters in 2014 and 2015, contact force-sensing catheters were used in 2016 and 2017 | iE33 and Epiq 7C (Philips Medical System, Andover, MA) systems equipped with S5-1 phased array and X7-2t matrix TEE transducers | 24 h Holter at 3, 6, 12 mo, q 12 mo thereafter | N/R |
| Szegedi 2022 | CCT, TEE | 256-slice scanner (Brilliance iCT 256, Philips Healthcare, Best, The Netherlands) | CARTO,(Biosense Webster, Inc., Diamond Bar, CA, USA) or ENSITE (St. Jude Medical, Inc., MN, USA), non-contact force-sensing catheters in 2014 and 2015, contact force-sensing catheters in 2016 and 2017 | iE33 and Epiq 7C (Philips Medical System, Andover, MA) systems equipped with S5-1 phased array and X7-2t matrix TEE transducers | 24 h Holter at 3, 6, 12 mo, q 12 mo thereafter | N/R |
| CCT= cardiac computed tomography, TEE= transesophageal echocardiography , TTE= transthoracic echocardiography, NA= not available, N/A= not applicable, N/R=not reported, mo=months, ECG=electrocardiogram, q=every, post-AFCA= after atrial fibrillation catheter ablation, w/= with, AAD= anti-arrhythmic drug | | | | | | |

**Supplementary Table 3:** Additional data on ablation methodology and left atrial appendage indices acquisition.

| **Author/**  **Year** | **AFCA type** | **AF type** | **LAA indices** | **LAA indices acquisition details** | **Timing of measurement** | **Ablation details** |
| --- | --- | --- | --- | --- | --- | --- |
| Tsao 2011 | RF | PAF, Per-AF | LAAEF | software developed by the Department of Biomedical  Engineering, Chung Yuan Christian University  (Chung-Li, Taiwan)  LAAEF= (max volume minus min volume)/max volume) | Pre-ablation* | WACA ± linear ablation  (LA roof, mitral isthmus)  Per-AF: stepwise ablation; WACA> linear ablation >CFAE |
| Park 2012 | RF | PAF, Per-AF | LAAV  LAAEF | 3D imaging processing workstation (Aquarius, Terarecon Inc., USA)  LAAEF = (maximum area -  minimum area)/maximum area) | 1 day pre-ablation | WACA  Per-AF: additional lines (mitral and cavotricuspid isthmus,  LA roof, anterior, posterior and septal line, superior vena cava), CFAEs |
| Machino-Ohtsuka  2013 | RF | Per-AF | LAAeV | Highest emptying velocity recorded with the sample volume positioned in proximity to the LAA orifice, average value of 3 consecutive beats in SR or 5 consecutive beats in AF | On the day of ablation | WACA ± linear ablation  (LA roof, superior vena cava, CFAEs) |
| Yoshida 2013 | RF | PAF, Per-AF | LAAeV | PAF patients underwent TEE during SR | Within 1 week pre-ablation | WACA |
| Combes 2013 | RF | Per-AF | LAAeV | average value of 10 consecutive  fibrillatory emptying waves | 2 days pre-ablation | Stepwise ablation (WACA >LA lines; roof; mitral isthmus; coronary sinus> CFAEs) |
| Kim 2014 | RF | Per-AF | LAAEF | LAAEF = (maximum area -  minimum area)/maximum area)  5 cardiac cycles were averaged | 1 day pre-ablation | WACA ± CFAEs |
| Gerede 2015 | CB | PAF | LAAeV | sample volume 1 cm into the orifice of the LAA, averaging five consecutive cardiac cycles | during cryoablation | PV isolation |
| Fukushima 2015 | RF | PAF | LAAeV | during SR, 4 mm sample volume  was positioned 1 cm from the LAA orifice, measured over three cardiac cycles | 2 days pre-ablation | WACA |
| Ariyama 2015 | RF | NPAF | LAAeV | average of 3 consecutive  cardiac cycles, sample volume at 5 mm from the orifice of the  LAA | Pre- ablation* | WACA ± additional lines  (LA roof, LA bottom, cavotricuspid isthmus, CFAEs, medial and lateral mitral isthmus) |
| Ma 2016 | RF | PAF, Per-AF | LAAeV | average value of 10 consecutive fibrillatory emptying waves, with the sample volume set at 1 cm inside the orifice of the LAA | 2 days pre-ablation | WACA |
| Nakatani 2016 | RF | PAF, Per-AF | LAAeV | NA | Pre- ablation* | WACA ± CFAEs |
| E Gul 2017 | RF | PeAF | LAAV  LAA_m | LAAV was calculated using GE Advantage Workstation 4.3  CW=bend in the proximal and middle part of the dominant lobe | Pre- ablation* | WACA |
| Zheng 2017 | RF | PAF, Per-AF | LAAV | NA | Pre-ablation* | WACA |
| Shiozawa 2017 | RF | PAF, Per-AF | LAAV  LAAeV  LAAOA  LAA_m | 3D workstation (ZIOSTATION2, Ziosoft, Tokyo, Japan)  LAAOA mas measured manually as the narrowest CSA  LAA_m was defined according to Di Base method | Pre-ablation* | WACA± additional lines  (LA roof, superior vena cava) |
| Pinto Teixeira 2017 | RF | PAF, Per-AF | LAAV | cardiac CTworkstation  (iNtuition, TeraRecon®) with volume-rendering software  Maximal LAAV measured at the end-systolic phase (usually 40% of the R-R interval), just before  the mitral valve opening, when the LA cavity was largest and  the LV cavity was smallest | Pre-ablation* | WACA ± additional lines  (mitral isthmus, LA roof, CFAEs) |
| Nedios 2017 | RF | PAF, Per-AF | LAAV | 3D volume rendering  (EnSite Verismo, MN,USA) | 1 day Pre-ablation during SR | WACA  Per-AF: additional box lesion (lines at mitral isthmus and posterior wall) |
| He 2018 | RF | PAF | LAAeV  LAAEF | GI-3DQ software  LAAEF = (LAAVmax − LAAVmin)/  LAAVmax × 100  If PAF at the time of TEE, measurements from ≥5 cardiac cycles were averaged | Pre-ablation* | N/A |
| Kocyigit 2019 | CB | PAF, Per-AF | LAAOA  LAA_m | CW: >4-cm long main lobe with a folded angle of<100° | Within 1 week pre-ablation | PV isolation |
| Du 2020 | RF | PAF, Per-AF | LAAV  LAAOA | NA | Pre-ablation* | WACA± linear ablation, CFAEs |
| Tian 2020 | RF | PAF, Per-AF | LAAV  LAAEF | Philips EBW4.5 workstation  LAAEF = (LAAVmax – LAAVmin)/LAAVmax × 100% | Pre-ablation* | WACA |
| Yang 2020 | RF | Per-AF | LAAeV | five cardiac cycles were averaged | Pre-ablation* | WACA+ linear ablation (tricuspid isthmus, mitral isthmus, posterior wall top or top plus bottom, anterior wall) |
| Wei 2020 | RF | PAF, Per-AF | LAAeV | Average value of 10 consecutive fibrillatory emptying waves | 1 day pre-ablation | WACA ± LA roofline and non-PV foci |
| Straube 2021 | CB | PAF, Per-AF | LAAV  LAAOA  LAA_m | EnSite Precision™  (Abbott Medical GmbH, Eschborn, Germany)  LAAV was computed automatically | Pre-ablation* | PV isolation |
| Gong 2021 | RF | PAF, Per-AF | LAAeV  LAA_m | CW: dominant lobe with an obvious bend in its proximal or middle part | Pre-ablation* | WACA  Per-AF: additional lines, CFAEs |
| Yang 2021 | RF | Per-AF | LAAeV | mean score of ten successive fibrillatory emptying waves | 1 day pre-ablation | WACA ± LA roofline and non-PV foci |
| You 2021 | RF, CB, RF+CB | PAF | LAAeV | 1 representative value when rhythm was stable, or by averaging the value of 5 consecutive sinus waves when rhythm was variable | Pre-ablation* | CB: PV isolation  RF: WACA |
| Istratoaie 2021 | RF | PAF | LAAeV | late diastolic positive outflow signal, measured using a 4 mm sample volume positioned at the entry of the LAA orifice  If PAF at the time of TEE, average value of five consecutive cardiac cycles | 1 day pre-ablation | WACA |
| Ma 2021 | RF | PAF, Per-AF | LAAeV | sample volume positioned at 1 cm from LAA orifice | Pre-ablation* | WACA  Per-AF: additional lines  (LA roof, superior vena cava) |
| Kielbasa 2021 | CB | PAF | LAAeV | sample volume positioned in the proximal one-third segment of the LAA , average value of three consecutive cardiac cycles in SR | On the day of ablation | PV isolation |
| Kim 2021 | RF | PAF, Per-AF | LAAV | multiplying the area of each  slice by its thickness and summing up the volumes of each slice  (semi-automatic software) | 1-2 days pre-ablation | WACA± CFAEs or linear ablation |
| Kim 2021 | RF | PAF, Per-AF | LAAeV | NA | 1-2 days pre-ablation | WACA± CFAEs or linear ablation |
| Spittler 2021 | RF | Per-AF | LAAeV | late diastolic emptying velocity  in SR and peak velocity in AF was taken in 30° within the first third of the LAA | On the day of ablation | Stepwise ablation (WACA>CFAEs> coronary sinus) |
| Simon 2022 | RF | PAF, Per-AF | LAAV  LAAeV  LAAOA | Semiautomated software  (EP Planning, Philips IntelliSpace Portal, Philips Healthcare, Best, The Netherlands) | CCT Pre-ablation*  TEE within 1 day pre-ablation | WACA  Per-AF: additional ablation lines |
| Szegedi 2022 | RF | PAF | LAA_m | Semiautomated software  (EP Planning, Philips IntelliSpace Portal, Philips Healthcare, Best, The Netherlands)  CW= dominant lobe has an obvious bend in the proximal or middle part | Pre-ablation* | WACA |
| SR= sinus rhythm, AF= atrial fibrillation, Per-AF= persistent atrial fibrillation, NPAF= non paroxysmal atrial fibrillation , PAF=paroxysmal atrial fibrillation, CCT=cardiac computed tomography, TEE= transesophageal echocardiography, AFCA= AF catheter ablation, RF= radiofrequency ablation, CB=cryoablation, NA= not available, CSA= cross sectional area, LAA= left atrial appendage, LAAV=LAA volume, LAAeV= LAA emptying velocity, LAAOA= LAA orfice area, LAAEF=LAA ejection fraction, LAA_m= LAA morphology, chicken wing versus non chicken wing, PV= pulmonary vein, WACA=wide area circular ablation, CFAE=complex fractionated atrial electrograms,*=no further specified, >=next step in ablation strategy. | | | | | | |

**Supplementary Table S4:** Fixed Effects model analysis and sensitivity analyses

| **LAA index** | **SMD or OR*** | **95% CI** | **p-value** |
| --- | --- | --- | --- |
| **LAAEF** | -0.61 | -0.80 ,-0.42 | <0.00001 |
| **LAAV** | 0.41 | 0.33,0.49 | <0.00001 |
| **LAA_m** | 1.19* | 0.91,1.57 | 0.21 |
| **LAAOA** | 0.34 | 0.24,0.45 | <0.00001 |
| **LAAeV** | -0.36 | -0.42,-0.31 | <0.00001 |

| **Sensitivity analysis; LAAEF** | | | | |
| --- | --- | --- | --- | --- |
| **Study Removed** | **SMD** | **95% CI** | **p-value** | **I^2^** |
| Tsao H et al 2011 | -0.74 | -1.15,-0.34 | 0.0003 | 73% |
| Park et al 2012 | -0.72 | -1.18,-0.27 | 0.002 | 74% |
| Kim et al 2014 | -0.75 | -1.17,-0.33 | 0.0005 | 70% |
| He et al 2018 | -0.58 | -0.96,-0.20 | 0.003 | 69% |
| Tian et al 2020 | -0.51 | -0.79,-0.24 | 0.0002 | 39% |

| **Sensitivity analysis; LAAV** | | | | |
| --- | --- | --- | --- | --- |
| **Study Removed** | **SMD** | **95% CI** | **p-value** | **I^2^** |
| Park et al 2012 | 0.55 | 0.38,0.72 | <0.00001 | 65% |
| Pinto Texteira et al 2017 | 0.48 | 0.33,0.64 | <0.00001 | 64% |
| Shiozawa et al 2017 | 0.50 | 0.34,0.66 | <0.00001 | 66% |
| Zheng et al 2017 | 0.48 | 0.33,0.63 | <0.00001 | 63% |
| E Gul et al 2017 | 0.53 | 0.36,0.69 | <0.00001 | 67% |
| Nedios et al 2017 | 0.53 | 0.36,0.70 | <0.00001 | 67% |
| Tian et al 2020 | 0.43 | 0.30,0.56 | <0.00001 | 44% |
| Du et al 2020 | 0.47 | 0.32,0.62 | <0.00001 | 61% |
| Kim et al 2021 | 0.56 | 0.36,0.75 | <0.00001 | 67% |
| Straube et 2021 | 0.55 | 0.37,0.73 | <0.00001 | 67% |
| Simon et al 2022 | 0.56 | 0.38,0.74 | <0.00001 | 64% |

| **Sensitivity analysis; LAA morphology (chicken wing versus no chicken wing)** | | | | |
| --- | --- | --- | --- | --- |
| **Study Removed** | **OR** | **95% CI** | **p-value** | **I^2^** |
| E Gul et al 2017 | 1.13 | 0.76,2.26 | 0.32 | 67% |
| Shiozawa et al 2017 | 1.37 | 0.80,2.34 | 0.25 | 65% |
| Kocyigit et al 2019 | 1.42 | 0.82,2.48 | 0.21 | 61% |
| Straube et al 2021 | 1.33 | 0.70,2.54 | 0.38 | 67% |
| Gong et al 2021 | 1.04 | 0.78,1.39 | 0.77 | 0% ** |
| Szegedi et al 2022 | 1.30 | 0.71,2.38 | 0.39 | 67% |

| **Sensitivity analysis; LAAOA** | | | | |
| --- | --- | --- | --- | --- |
| **Study Removed** | **SMD** | **95% CI** | **p-value** | **I^2^** |
| Shiozawa et al 2017 | 0.33 | 0.17,0.49 | <0.00001 | 47% |
| Kocyigit et al 2019 | 0.39 | 0.36,0.52 | <0.00001 | 9% ** |
| Du et al 2020 | 0.32 | 0.18,0.47 | <0.00001 | 37% |
| Straube et al 2021 | 0.39 | 0.19,0.58 | <0.00001 | 43% |
| Simon et al 2022 | 0.30 | 0.13,0.48 | 0.0007 | 29% |
| **Sensitivity analysis ; LAAeV** | | | | |
| **Study Removed** | **SMD** | **95% CI** | **p-value** | **I^2^** |
| Yoshida et al 2013 (per-AF) | -0.56 | -0.73,-0.40 | <0.00001 | 83% |
| Yoshida et al 2013 (PAF) | -0.57 | -0.73,-0.40 | <0.00001 | 83% |
| Combes et al 2013 | -0.55 | -0.71,-0.39 | <0.00001 | 83% |
| Gerede et al 2015 | -0.54 | -0.70,-0.38 | <0.00001 | 82% |
| Fukushima et al 2015 | -0.57 | -0.74,-0.40 | <0.00001 | 83% |
| Ariyama et al 2015 | -0.57 | -0.73,-0.40 | <0.00001 | 83% |
| Nakatani et al 2016 | -0.59 | -0.76,-0.43 | <0.00001 | 83% |
| Ma et al 2017 paf | -0.54 | -0.70,-0.38 | <0.00001 | 82% |
| Ma et al 2017 per-af | -0.52 | -0.68,-0.36 | <0.00001 | 80% |
| Shiozawa et al 2017 | -0.59 | -0.75,-0.42 | <0.00001 | 83% |
| He et al 2018 | -0.54 | -0.70,-0.38 | <0.00001 | 82% |
| Spittler et al 2019 | -0.56 | -0.73,-0.40 | <0.00001 | 83% |
| Wei et al 2020 | -0.57 | -0.74,-0.40 | <0.00001 | 83% |
| Yang et al 2020 | -0.56 | -0.73,-0.39 | <0.00001 | 83% |
| You et al 2021 | -0.57 | -0.74,-0.40 | <0.00001 | 83% |
| Kim et al w/o ER 2021 | -0.60 | -0.76,-0.43 | <0.00001 | 79% |
| Kim et al with ER 2021 | -0.59 | -0.78,-0.40 | <0.00001 | 83% |
| Istratoaie et al 2021 | -0.52 | -0.67,-0.36 | <0.00001 | 80% |
| Kielbasa et al 2021 | -0.58 | -0.75,-0.41 | <0.00001 | 83% |
| Gong et al 2021 | -0.58 | -0.75,-0.42 | <0.00001 | 83% |
| Yang et al 2021 | -0.56 | -0.73,-0.40 | <0.00001 | 83% |
| Ma et al 2021 | -0.55 | -0.71,-0.38 | <0.00001 | 82% |
| Simon et al 2022 | -0.60 | -0.76,-0.43 | <0.00001 | 80% |
| LAA= left atrial appendage, LAAV=LAA volume, LAAeV= LAA emptying velocity, LAAOA= LAA orfice area, LAAEF=LAA ejection fraction, LAA_m= LAA morphology, chicken wing versus non chicken wing, CI= confidence intervals, SMD= standardized mean difference, OR=odds ratio | | | | |

**Supplementary Table S5**: Study quality according to Newcastle-Ottawa scale (NOS). Studies were defined as high quality if they had more than seven points, as medium quality if they had between four and six points, and as poor quality if they had fewer than four points.

| **Author/**  **Year** | **Journal** | **Selection** | **Comparability** | **Outcome** | **Total Score (quality)** |
| --- | --- | --- | --- | --- | --- |
| Tsao 2011 | Am J Cardiol | 3 | 0 | 2 | 5 (moderate) |
| Park 2012 | Int J Cardiovasc Imaging | 4 | 0 | 3 | 7 (high) |
| Machino-Ohtsuka  2013 | Circ J | 4 | 0 | 2 | 6 (moderate) |
| Yoshida 2013 | Echocardiography | 2 | 0 | 2 | 4 (moderate) |
| Combes 2013 | Arch Cardiovasc Dis | 4 | 0 | 2 | 6 (moderate) |
| Kim 2014 | Int Heart J | 4 | 0 | 3 | 7 (high) |
| Gerede 2015 | Anatol J Cardiol | 2 | 0 | 2 | 4 (moderate) |
| Fukushima 2015 | Echocardiography | 3 | 0 | 3 | 6 (moderate) |
| Ariyama 2015 | Echocardiography | 4 | 0 | 3 | 7 (high) |
| Ma 2016 | J Interv Card Electrophysiol | 4 | 0 | 3 | 7 (high) |
| Nakatani 2016 | J Cardiovasc Electrophysiol | 4 | 0 | 3 | 7 (high) |
| E Gul 2017 | J Atr Fibrillation | 4 | 0 | 2 | 6 (moderate) |
| Zheng 2017 | Zhonghua Xin Xue Guan Bing Za Zhi | 4 | 0 | 2 | 6 (moderate) |
| Shiozawa 2017 | Cardiol Res | 4 | 0 | 3 | 7 (high) |
| Pinto Teixeira 2017 | J Interv Card Electrophysiol | 2 | 0 | 2 | 4 (moderate) |
| Nedios 2017 | Europace | 3 | 0 | 3 | 6 (moderate) |
| He 2018 | Echocardiography | 3 | 0 | 2 | 5 (moderate) |
| Kocyigit 2019 | J Cardiovasc Comput Tomogr | 4 | 0 | 3 | 7 (high) |
| Du 2020 | J Electrocardiol | 3 | 0 | 2 | 5 (moderate) |
| Tian 2020 | Sci Rep | 4 | 0 | 2 | 6 (moderate) |
| Yang 2020 | Clin Cardiol | 3 | 0 | 2 | 5 (moderate) |
| Wei 2020 | Mediators Inflamm | 4 | 0 | 2 | 6 (moderate) |
| Straube 2021 | Clin Cardiol | 4 | 0 | 3 | 7 (high) |
| Gong 2021 | Front Cardiovasc Med | 2 | 0 | 2 | 4 (moderate) |
| Yang 2021 | J Thorac Dis | 4 | 0 | 3 | 7 (high) |
| You 2021 | Front Cardiovasc Med | 4 | 0 | 3 | 7 (high) |
| Istratoaie 2021 | Diagnostics (Basel) | 4 | 0 | 3 | 7 (high) |
| Ma 2021 | Open Heart | 3 | 0 | 2 | 5 (moderate) |
| Kielbasa 2021 | Kardiol Pol | 4 | 0 | 3 | 7 (high) |
| Kim 2021 | Int J Cardiovasc Imaging | 4 | 0 | 3 | 7 (high) |
| Kim 2021 | JACC Clin Electrophysiol | 4 | 0 | 3 | 7 (high) |
| Spittler 2021 | J Cardiovasc Electrophysiol | 4 | 0 | 3 | 7 (high) |
| Simon 2022 | Clin Cardiol | 4 | 0 | 3 | 7 (high) |
| Szegedi 2022 | Front Cardiovasc Med | 4 | 0 | 3 | 7 (high) |

**Supplementary Figure 1:** Subgroup analysis comparing mean difference in LAA volume between arrhythmia recurrence and arrhythmia free patients, separately conducted for studies exploring pulmonary vein isolation only (PVI) and PVI plus additional ablation lines.

**
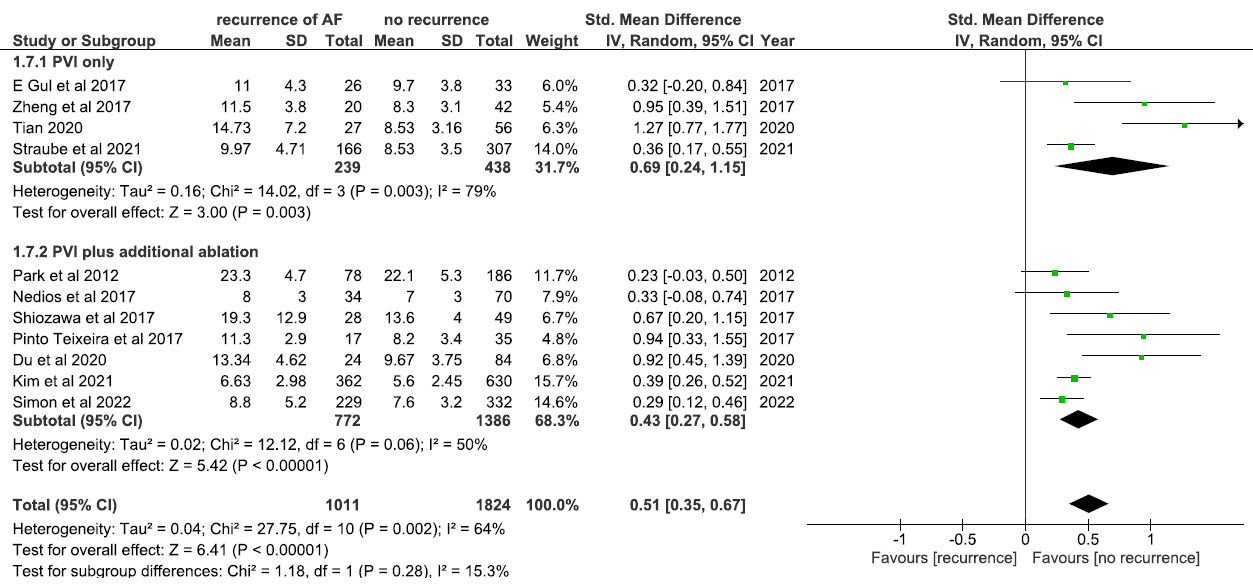
**

**Supplementary Figure 2:** Subgroup analysis comparing Chicken wing versus non-chicken wing LAA morphology between arrhythmia recurrence and arrhythmia free patients, separately conducted for studies exploring pulmonary vein isolation only (PVI) and PVI plus additional ablation lines.


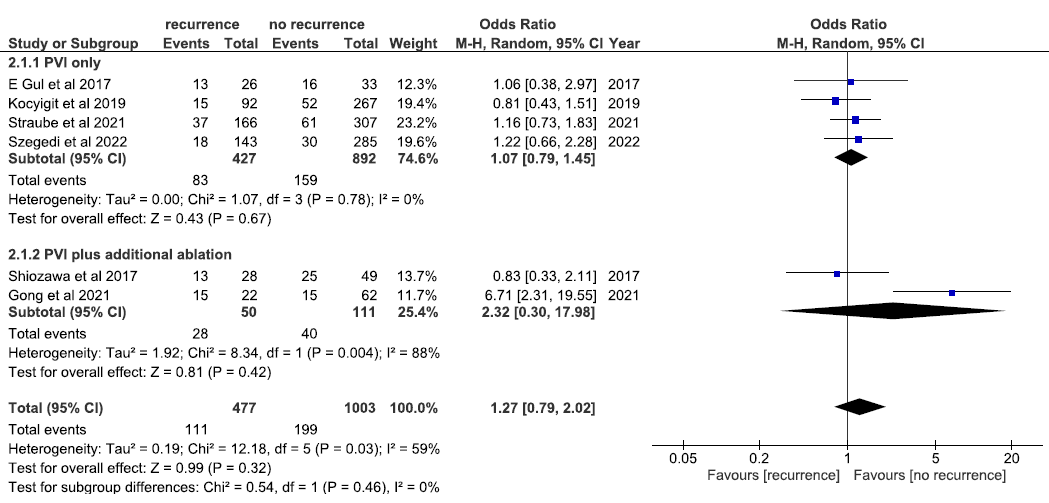


**Supplementary Figure 3:** Subgroup analysis comparing LAA emptying velocity between arrhythmia recurrence and arrhythmia free patients, separately conducted for studies exploring pulmonary vein isolation only (PVI) and PVI plus additional ablation lines.


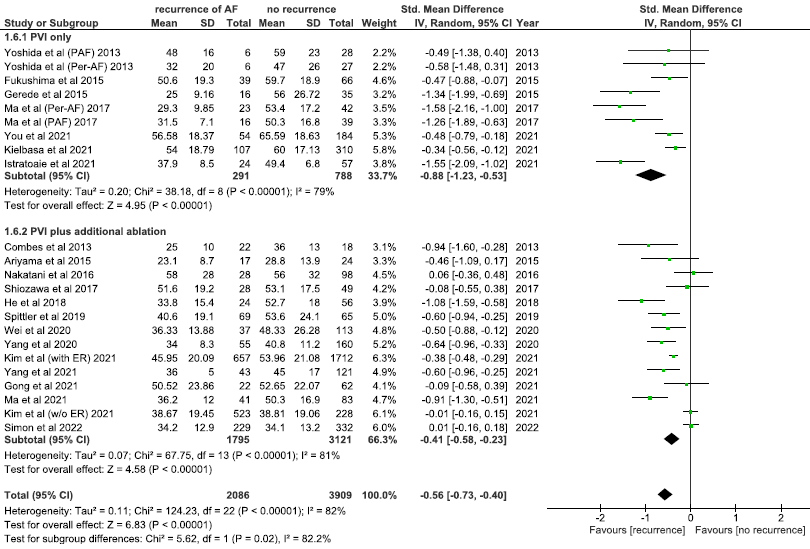


**Supplementary Figure 4:** Subgroup analysis comparing LAA emptying velocity between arrhythmia recurrence and arrhythmia free patients, separately conducted for studies exploring paroxysmal, persistent and mixed AF populations.


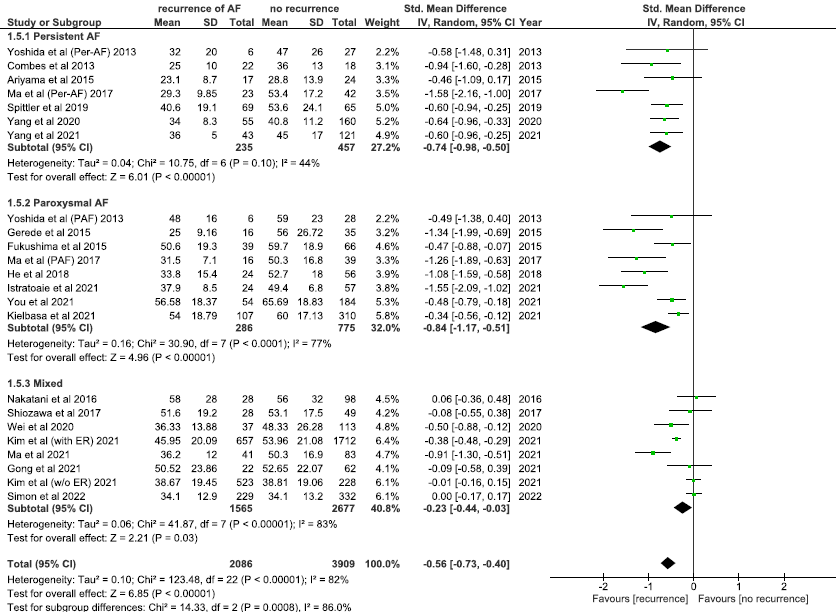


**Supplementary Figure 5**: Funnel plot for LAA volume in arrhythmia recurrence and arrhythmia free groups.


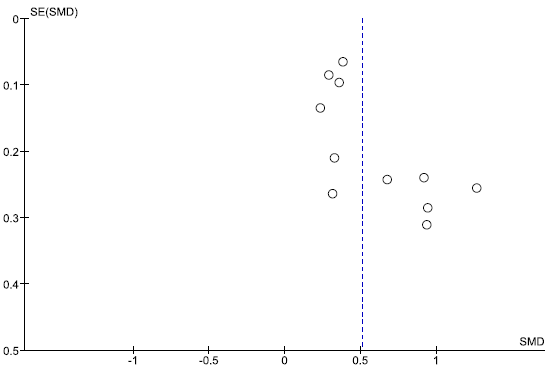


**Supplementary Figure 6**: Funnel plot for LAA emptying velocity in arrhythmia recurrence and arrhythmia free groups.


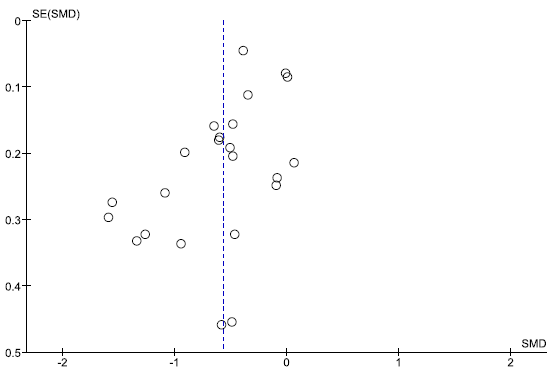

Supplement: Supplementary file 1 — Additional file 1. [file 43044_2023_356_MOESM1_ESM.docx]
